# Phenome-wide Mendelian-randomization study of genetically determined vitamin D on multiple health outcomes using the UK Biobank study

**DOI:** 10.1093/ije/dyz182

**Published:** 2019-09-13

**Authors:** Xiangrui Meng, Xue Li, Maria N Timofeeva, Yazhou He, Athina Spiliopoulou, Wei-Qi Wei, Aliya Gifford, Hongjiang Wu, Timothy Varley, Peter Joshi, Joshua C Denny, Susan M Farrington, Lina Zgaga, Malcolm G Dunlop, Paul McKeigue, Harry Campbell, Evropi Theodoratou

**Affiliations:** 1 Centre for Global Health Research, Usher Institute of Population Health Sciences and Informatics, University of Edinburgh, Edinburgh, UK; 2 Colon Cancer Genetics Group and Academic Coloproctology, Institute of Genetics and Molecular Medicine, University of Edinburgh, and MRC Human Genetics Unit Western General Hospital Edinburgh, Edinburgh, UK; 3 Edinburgh Cancer Research Centre, MRC Institute of Genetics and Molecular Medicine, University of Edinburgh, Edinburgh, UK; 4 West China School of Medicine, West China Hospital, Sichuan University, Chengdu, Sichuan Province, P. R. China; 5 Centre for Population Health Sciences, Usher Institute of Population Health Sciences and Informatics, University of Edinburgh, Edinburgh, Scotland, UK; 6 Department of Biomedical Informatics, Vanderbilt University Medical Centre, Nashville, TN, USA; 7 Public Health and Intelligence, NHS National Services Scotland, Edinburgh, UK; 8 Discipline of Public Health and Primary Care, Institute of Population Health, Trinity College Dublin, University of Dublin, Dublin, Ireland

**Keywords:** 25(OH)D, vitamin D, PheWAS, Mendelian randomization

## Abstract

**Background:**

Vitamin D deficiency is highly prevalent across the globe. Existing studies suggest that a low vitamin D level is associated with more than 130 outcomes. Exploring the causal role of vitamin D in health outcomes could support or question vitamin D supplementation.

**Methods:**

We carried out a systematic literature review of previous Mendelian-randomization studies on vitamin D. We then implemented a Mendelian Randomization–Phenome Wide Association Study (MR-PheWAS) analysis on data from 339 256 individuals of White British origin from UK Biobank. We first ran a PheWAS analysis to test the associations between a 25(OH)D polygenic risk score and 920 disease outcomes, and then nine phenotypes (i.e. systolic blood pressure, diastolic blood pressure, risk of hypertension, T2D, ischaemic heart disease, body mass index, depression, non-vertebral fracture and all-cause mortality) that met the pre-defined inclusion criteria for further analysis were examined by multiple MR analytical approaches to explore causality.

**Results:**

The PheWAS analysis did not identify any health outcome associated with the 25(OH)D polygenic risk score. Although a selection of nine outcomes were reported in previous Mendelian-randomization studies or umbrella reviews to be associated with vitamin D, our MR analysis, with substantial study power (>80% power to detect an association with an odds ratio >1.2 for per standard deviation increase of log-transformed 25[OH]D), was unable to support an interpretation of causal association.

**Conclusions:**

We investigated the putative causal effects of vitamin D on multiple health outcomes in a White population. We did not support a causal effect on any of the disease outcomes tested. However, we cannot exclude small causal effects or effects on outcomes that we did not have enough power to explore due to the small number of cases.

**Key Messages**

- Observational studies have identified associations between vitamin D levels and hundreds of disease outcomes. However, evidence of causality for most of these associations is either lacking or not confirmed by randomized clinical trials or Mendelian-randomization studies.
- With the Mendelian Randomization–Phenome Wide Association Study design, we explored the causal relationships between vitamin D level and 920 outcomes in the UK Biobank cohort. None of these outcomes was causally associated with vitamin D at a moderate or higher effect size.
- Small causal effects that we did not have enough power to explore in the present study could be studied in the future with larger sample sizes to achieve sufficient statistical power.

## Introduction

Vitamin D status is an important public-health issue due to the high prevalence of vitamin D insufficiency and deficiency worldwide.^1^ Furthermore, it has been reported to be associated with many non-skeletal outcomes (e.g. cardiovascular disease, cognitive impairment and cancer).^2^ In our recent umbrella review of meta-analyses of randomized clinical trials (RCTs) and of observational studies, we have found that serum 25-hydroxyvitamin D (25(OH)D) or supplemental vitamin D has been linked to more than 130 unique health outcomes.^3^ However, the majority of the studies yielded conflicting results and no association was convincing.^3^

With large cohorts linked to electronic medical records (EMRs), the Phenome Wide Association Study (PheWAS) design has been proposed as a high-throughput approach to comprehensively evaluate associations between genetic variants and a wide range of phenotypes (usually generated by EMR). The PheWAS method has been proven to be useful in the replication of hundreds of known genotype–phenotype associations as well the identification of new associations.^4^ Since phenotypes defined by EMRs are largely correlated, Bonferroni correction for a conventional PheWAS using general linear models is over-conservative. Thus, a novel Bayesian analysis framework, termed TreeWAS, has been developed.^5^ TreeWAS is shown to increase statistical power by up to 20% and can detect new hits missed by a conventional PheWAS.^5^

In traditional epidemiological analysis, a causal effect of 25(OH)D on disease outcomes cannot be unambiguously demonstrated due to the known limitations of observational research including unmeasured confounding factors and reverse causality. By using single-nucleotide polymorphisms (SNPs) associated with 25(OH)D in an instrumental variable (IV) analysis, also known as Mendelian-randomization (MR) analysis, we can largely overcome these limitations.

In the current study, we first conducted a systematic literature review of previous MR studies on 25(OH)D. Then, we analysed 339 256 individuals from the UK Biobank study by implementing a conventional PheWAS and the Bayesian TreeWAS for a large number of health outcomes based on linked EMRs. Finally, for a selection of outcomes based on the results from PheWAS/TreeWAS, power and evidence from previous studies, we performed further MR analysis.

## Methods

### Systematic literature review for MR studies on 25(OH)D

**Figure 1:**
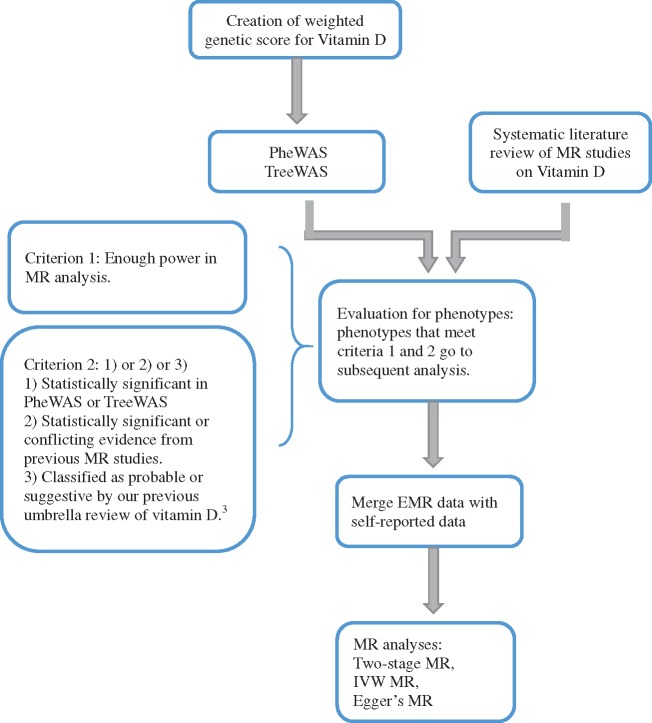
Study flowchart.

The main steps of the study are presented in Figure 1. First, we carried out a systematic literature review of all published MR studies exploring the causal effect of 25(OH)D levels on any outcome. The Medline and Embase databases were searched for up to 1 May 2019. For details on the search strategy and search algorithm, please see Supplementary Table 1, available as Supplementary data at *IJE* online. Any review articles, non-English articles or conference abstracts were excluded. Studies on the impact of other markers/exposures on 25(OH)D levels were also excluded. References from the included studies were checked to identify any additional relevant studies. The literature search and review process were done in parallel by two authors (X.M. and Y.H.). Data extraction was done by X.M. and then confirmed by Y.H.

### Study population

UK Biobank is a very large population-based prospective study established to allow detailed investigations of genetic and non-genetic determinants of the diseases in middle- and old-aged adults.^6^ More than 500 000 individuals were recruited between 2006 and 2010, all of whom gave written consent and underwent baseline measurements, including questionnaires, interview, anthropometric and clinical measurements. Participants donated blood samples for genotyping and biomarker analysis. In addition, UK Biobank participants were linked to their EMR data, including hospital inpatient, cancer-registry and death-registry data.

In this study, we used the UK Biobank genetic data of 488 378 participants. Genetic quality control was done centrally by UK Biobank.^7^ A total of 339 256 unrelated White British individuals were included in our final analysis (Supplementary Methods, available as Supplementary data at *IJE* online).

### Statistical analysis

We implemented all statistical analyses using R 3.3.2. We used the R package developed by Carroll *et al.* for the PheWAS analysis.^8^ We used the R package developed by Cortes *et al.* for the TreeWAS analysis.^5^

#### Creation of 25(OH)D genetic-risk score

We created a genetic-risk score for 25(OH)D. In the selection of variants, we used the results from the largest genome-wide association study (GWAS) on 25(OH)D in a White population with a total of 79 336 individuals of European ancestry (the SUNLIGHT GWAS).^9^ The SUNLIGHT GWAS identified six independent loci associated with serum 25(OH)D concentration that explained 2.84% of the trait variance: rs3755967 (*GC*), rs12785878 (*NADSYN1/DHCR7*), rs10741657 (*CYP2R1*), rs17216707 (*CYP24A1*), rs10745742 (*AMDHD1*) and rs8018720 (*SEC23A*). We created a genetic score by adding the number of effect alleles carried in each of the six SNPs and weighted based on their effect estimates from the SUNLIGHT GWAS.

#### PheWAS/TreeWAS analysis

The association between the genetic-risk score and common confounders was first tested. In the PheWAS analysis, we only included disease groups with more than 200 cases, as suggested by a simulation of power estimates for PheWAS analysis.^10^ We then applied logistic-regression adjusting for gender, age, body mass index (BMI), the UK Biobank assessment centre attended, east and north co-ordinates of home address, and the first five ancestral principal components. A total of 920 disease phenotypes were tested and a *P*-value of <5.44 × 10^–5^ was regarded as statistically significant based on Bonferroni correction (0.05/920 = 5.44 × 10^–5^).

We also applied the TreeWAS Bayesian analysis. The case–control status of participants was defined by their ICD10 codes (from hospital episode, cancer-registry and death-registry data). Due to the complexity of converting ICD9 codes into ICD10 codes, records of ICD9 were discarded. The same weighted genetic-risk score was employed to test the association between the score and all ICD10 codes presented in UK Biobank data. In our TreeWAS analysis, nodes with posterior probability (PP) of a non-zero effect >0.75 were considered as significant.^5^

#### MR analysis

For those phenotypes for which there was enough statistical power for a MR study (>80%) and (i) were statistically significant in the aforementioned PheWAS/TreeWAS analysis or (ii) were classified as probable or suggestive in our previous umbrella review^3^ or (iii) were found to be statistically significant or with conflicting evidence in previous MR studies, we implemented MR analysis to further control for bias and assess the causality of the observed associations. To increase the statistical power, we used UK Biobank self-reported medical conditions to include cases that were not captured by EMR data. With power calculation, we estimated that we had 80% power to detect a causal odds ratio (OR) of 1.2 for outcomes with more than 9977 cases assuming that the genetic instrument explains 2.84% of the variance of 25(OH)D levels and that the case:control ratio was 1:5 or larger at alpha = 0.05 level.^11^ For these outcomes, we then ran MR analyses using multiple methods: (i) two-stage MR, (ii) inverse variance weighted (IVW) MR and (iii) Egger’s regression MR (Supplementary Methods, available as Supplementary data at *IJE* online). In addition, we constructed the identical genetic-risk score in 2821 control individuals of the Study of Colorectal Cancer in Scotland (SOCCS) (see Supplementary Methods, available as Supplementary data at *IJE* online, for a description of the SOCCS study) to estimate the variance of 25(OH)D levels explained by this score and the corresponding *F*-statistics, given that we did not have individual 25(OH)D-level data from the UK Biobank.

## Results

### Systematic literature review of 25(OH)D MR studies

After applying our inclusion and exclusion criteria, 63 MR studies were included in our systematic literature review (Supplementary Figure 1, available as Supplementary data at *IJE* online). The causal effect of vitamin D has been examined across a range of disease outcomes and a causal role of vitamin D is not supported for the majority of them. Disease outcomes that were ever reported to be causally associated with vitamin D levels include type 2 diabetes (T2D), total adiponectin, diastolic blood pressure (DBP), risk of hypertension, multiple sclerosis, Alzheimer’s disease, all-cause mortality, cancer mortality, mortality excluding cancer and cardiovascular events, ovarian cancer, HDL-cholesterol, triglycerides, high-density lipoprotein, delirium and cognitive functions. However, for some of these outcomes, the evidence across different MR studies is not consistent. Detailed results of the systematic review of previous MR studies are present in Supplementary Methods, available as Supplementary data at *IJE* online, and Supplementary Table 2, available as Supplementary data at *IJE* online.

### Descriptive analysis of the included UK Biobank participants

We included 339 256 British White unrelated individuals from the UK Biobank cohort, 53.68% of whom were female. All six SNPs satisfied the Hardy-Weinberg equilibrium test (Table 1 and Supplementary Table 3, available as Supplementary data at *IJE* online). The association between the score of six SNPs and common confounding factors is presented in Table 2. Except for the UK Biobank assessment centre (*P *=* *1.30 × 10^–17^), all other confounding factors were not associated with the score. We then tested the associations between the SNPs and the geographic regions (England and Wales vs Scotland) and found that two SNPs (rs12785878 and rs10745742) were unevenly distributed across the UK. Since we have adjusted for the assessment centre and latitude as covariates, we did not expect the association between score and assessment centre to bias our results.

**Table 1.** Demographic characteristics of the UK Biobank participants and genotype counts of the six SNPs included in the genetic-risk score

Variable	Value
**Demographic characteristics (*n* = 339 256)**	
Female	182 110 (53.68%)
Age	56.89 (7.99) years
BMI	27.40 (4.76) kg/m^2^
**Genotype counts**	
rs3755967 polymorphism (*n*=338 753)	
CC	169 710 (50.10%)
CT	140 206 (41.39%)
TT	28 837 (8.51%)
Hardy-Weinberg test *P*-value	0.52
rs10741657 polymorphism (*n* = 339 256)	
AA	55 617 (16.39%)
AG	163 064 (48.07%)
GG	120 575 (35.54%)
Hardy-Weinberg test *P*-value	0.83
rs12785878 polymorphism (*n* = 339 256)	
TT	211 627 (62.38%)
TG	112 585 (33.19%)
GG	15 044 (4.43%)
Hardy-Weinberg test *P*-value	0.35
rs10745742 polymorphism (*n* = 336 987)	
TT	47 797 (14.18%)
TC	158 392 (47.00%)
CC	130 798 (38.82%)
Hardy-Weinberg test *P*-value	0.29
rs8018720 polymorphism (*n* = 339 256)	
GG	10 666 (3.14%)
GC	98 435 (29.02%)
CC	230 155 (67.84%)
Hardy-Weinberg test *P*-value	0.80
rs17216707 polymorphism (*n* = 324 016)	
TT	216 735 (66.89%)
TC	96 403 (29.75%)
CC	10 878 (3.36%)
Hardy-Weinberg test *P*-value	0.78

Continuous variables are presented as mean (standard deviation), whereas categorical variables are presents as *N* (%).

BMI, body mass index.

**Table 2.** Association of the instrumental variable (weighted genetic-risk score) with potential confounding factors

	Continuous		Categorical	
Confounding factors	Beta (SE)	*P*-value	*F*-value	*P*-value
Age	0.156 (0.202)	0.441		
BMI	0.224 (0.121)	0.063		
Time spend outdoors in summer	–0.077 (0.091)	0.394		
Time spend outdoors in winter	0.083 (0.119)	0.485		
Sex			0.455	0.500
Assessment centre			6.164	1.30 × 10^–17a^
Average household income before tax			1.213	0.296
Qualification			0.490	0.843
Alcohol intake frequency			1.419	0.203

Univariate linear regression was conducted for continuous confounding factors and analysis of variance was conducted for categorical factors.

a *P *<* *0.05.

### PheWAS and TreeWAS

**Figure 2:**
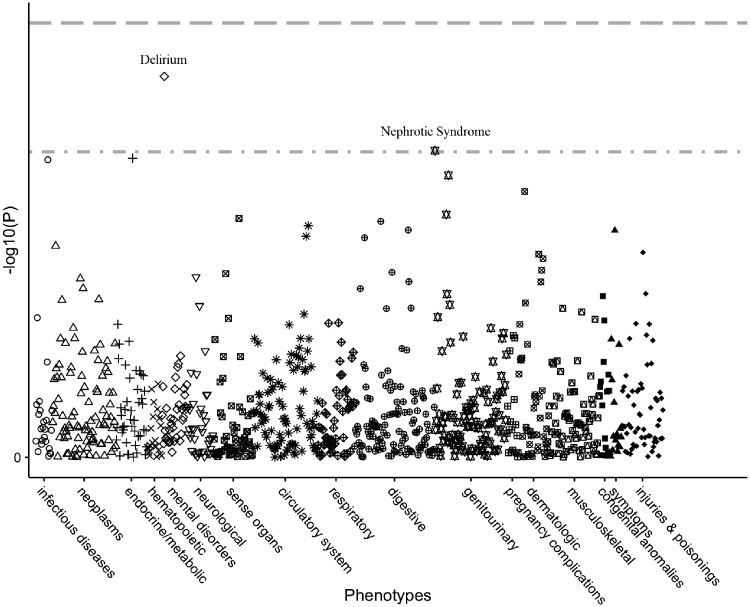
Manhattan plot for results of PheWAS analysis. Phenotypes aggregated on International Classification of Disease codes were plotted with the –log10 *P*-value of each association. The first line indicates a Bonferroni-corrected *P*-level of 5.44 × 10^–5^ and the second line indicates a *P*-level of 0.001. No phenotype survived Bonferroni correction. There were only two phenotypes with a *P*-value <0.001, which were delirium (*P *=* *1.83 × 10^–4^) and nephrotic syndrome (*P *=* *9.75 × 10^–4^).

In the conventional PheWAS, we tested associations between the score and 920 outcomes (>200 cases). No associations survived the Bonferroni multiple-testing correction. There were only two phenotypes with a *P*-value <0.001 that were reported as suggestive associations, including delirium (517 cases, *P *=* *1.83 × 10^–4^) and nephrotic syndrome (374 cases, *P *=* *9.75 × 10^–4^) (Figure 2 and Supplementary Table 4, available as Supplementary data at *IJE* online). The *P*-value for the association between the score and vitamin D deficiency was 0.00116 (291 cases), which was the third smallest *P*-value among all tested associations. We additionally performed a sensitivity analysis without adjustment for BMI (Supplementary Table 5, available as Supplementary data at *IJE* online) and the results showed no difference in the significance of PheWAS associations when compared to that with adjustment for BMI (Supplementary Table 4, available as Supplementary data at *IJE* online).

In the TreeWAS, the largest PP for all tested phenotypes was 0.26, which was for the calculus of the ureter (ICD10 code, N20.1). Since a PP >0.75 was needed to take findings from the TreeWAS forward, there were no putative associations found from the TreeWAS analysis.

### MR analysis

Based on our eligibility criteria, the following outcomes were selected to be further examined using MR: systolic blood pressure (SBP), DBP, risk of hypertension, risk of T2D, risk of ischaemic heart disease (IHD), BMI, risk of depression, risk of non-vertebral fracture and all-cause mortality. Incorporating self-reported data increased the numbers of cases for some of the outcomes (Table 3). We first applied linear regression between the genetic-risk score and measured plasma 25(OH)D levels in the controls from the SOCCS study (*N* = 2821). The *R*^2^ value was 1.61% and the *F*-statistic was 45.96, indicating that the genetic-risk score is a strong IV for our MR analysis.^12^ We then applied the second stage of the MR analysis and we did not observe any causal effects for all the tested outcomes. Results from the three different MR methods were consistent (Table 4). Additionally, for the MR Egger’s regression, the *P*-value of the intercept term for all outcomes was >0.05, indicating no evidence of unbalanced pleiotropy (Supplementary Table 6, available as Supplementary data at *IJE* online) among the variants we used.

**Table 3.** Number of cases in Mendelian-randomization analysis

Outcomes	*N* total^a^	*N*, EMR^b^	*N*, SR^c^	*N*, both^d^
SBP^e^	319 778	NA	NA	NA
DBP^e^	319 779	NA	NA	NA
Hypertension	106 405	16 905 (15.9%)	42 317 (39.8%)	47 183 (44.3%)
T2D	15 958	13 692 (85.8%)	671 (4.2%)	1595 (10.0%)
IHD	28 337	13 062 (46.1%)	2556 (9.0%)	12 719 (44.9%)
BMI^e^	338 172	NA	NA	NA
Depression	23 294	5382 (23.1%)	13 628 (58.5%)	4284 (18.4%)
Non-vertebral fracture	23 603	15 811 (67.0%)	6382 (27.0%)	1410 (6.0%)
All-cause mortality	9830	9830 (100%)	NA	NA

EMR, electronic medical records; SR, self-reported medical conditions; SBP, systolic blood pressure; DBP, diastolic blood pressure; T2D, type 2 diabetes; IHD, ischaemic heart disease; BMI, body mass index.

a Total number of cases.

b Number of cases captured by EMR data only.

c Number of cases captured by SR data only.

d Number of cases captured by both EMR and SR.

e Continuous variable, data come from baseline anthropometric measurement data.

**Table 4.** Mendelian-randomization causal-effect estimates for nine selected outcomes

Method	beta	se	*P*-value	OR	95% CI	*N* total/*N* cases	Power
**Systolic blood pressure**						319 778	NA
Two-stage MR	–0.669	0.449	0.137	NA	NA		
IVW MR	–0.648	0.451	0.210	NA	NA		
Egger's regression	–0.180	1.086	0.876	NA	NA		
**Diastolic blood pressure**						319 779	NA
Two-stage MR	–0.121	0.251	0.629	NA	NA		
IVW MR	–0.117	0.251	0.661	NA	NA		
Egger's regression	0.491	0.530	0.407	NA	NA		
**Hypertension**						339 256/106 405	1.00/0.99
Two-stage MR	–0.056	0.059	0.343	0.976	0.928–1.026		
IVW MR	–0.063	0.060	0.340	0.973	0.911–1.040		
Egger's regression	0.084	0.175	0.657	1.037	0.841–1.278		
**Type 2 Diabetes**						339 256/15 958	0.97/0.51
Two-stage MR	–0.060	0.126	0.632	0.974	0.876–1.083		
IVW MR	–0.067	0.126	0.617	0.971	0.845–1.117		
Egger's regression	0.242	0.244	0.377	1.110	0.829–1.485		
**Ischaemic heart disease**						339 256/28 337	1.00/0.74
Two-stage MR	0.049	0.096	0.611	1.021	0.942–1.107		
IVW MR	0.047	0.096	0.647	1.020	0.917–1.135		
Egger's regression	0.109	0.219	0.645	1.048	0.807–1.360		
**Body mass index**						338 172	NA
Two-stage MR	0.128	0.120	0.288	NA	NA		
IVW MR	0.130	0.121	0.329	NA	NA		
Egger's regression	–0.099	0.213	0.665	NA	NA		
**Depression**						339 256/23 294	0.99/0.66
Two-stage MR	–0.216	0.102	0.034	0.911	0.837–0.993		
IVW MR	–0.212	0.102	0.093	0.913	0.816–1.022		
Egger's regression	–0.311	0.180	0.158	0.875	0.706–1.084		
**Non-vertebral fracture**						339 256/23 603	1.00/0.66
Two-stage MR	–0.068	0.101	0.497	0.971	0.892–1.057		
IVW MR	–0.074	0.101	0.495	0.969	0.867–1.083		
Egger's regression	–0.092	0.265	0.747	0.961	0.700–1.320		
**All-cause mortality**						339 256/9830	0.85/0.35
Two-stage MR	0.073	0.154	0.634	1.032	0.907–1.175		
IVW MR	0.069	0.154	0.671	1.030	0.869–1.222		
Egger's regression	0.192	0.272	0.520	1.086	0.785–1.503		

MR effect estimates were done with three different MR methods. OR was calculated as exponential of beta × SD [the standard deviation (SD) of the log-transformed 25(OH)D level in an independent British population, SOCCS, which was 0.430], whose unit was per SD increase in log-transformed 25(OH)D levels. The upper/lower 95% CI was calculated similarly; with the same unit as the OR. Power was calculated assuming a *R*^2^ value of 0.0284, OR of 1.2/1.1 and significance level at 0.05.

MR, Mendelian randomization; IVW, inverse variance weighted; OR, odds ratio.

## Discussion

### Main findings

In order to investigate the safety and rationale of population-wide measurements to raise vitamin D levels (such as fortification of staple food with vitamin D), we conducted a high-throughput PheWAS/TreeWAS study on more than 920 outcomes in a UK Biobank sample of 339 256 British White individuals, followed by MR analyses for selected outcomes. The large sample size ensured the study was well powered to detect moderate to large causal effects (OR >1.2) for outcomes with more than 9997 cases.

The PheWAS/TreeWAS analysis used a weighted genetic score of six SNPs as a proxy of the genetically determined 25(OH)D level and examined its association across a wide range of disease outcomes. In this initial scan, we did not observe any significant associations with adjustment for a number of covariates. Additionally, given that there is much debate in the literature suggesting not to adjust for heritable covariates in genome-wide association studies,^13^ we performed a sensitivity analysis without BMI as a covariate. There were some differences in the actual effect sizes, but the direction and statistical significance of the PheWAS associations were consistent between the two models.

We then integrated EMR data with self-reported medical conditions for the final MR analysis and explored the causal effect of vitamin D on SBP, DBP, risk of hypertension, T2D, IHD, BMI, risk of depression, non-vertebral fracture and all-cause mortality. This merged dataset increased the number of cases and re-assigned spurious controls, thus increasing the study power. For outcomes tested in MR, we had statistical power of nearly 100% for detecting a true effect of or larger than 1.2, except for all-cause mortality (85% power). The estimated causal-effect sizes for all outcomes were close to null with narrow confidence intervals, which suggested that there is no moderate to large causal effect of 25(OH)D on the nine tested outcomes.

Among these nine outcomes, associations between vitamin D and blood pressure, hypertension, T2D, IHD and BMI were suggestive from previous observational studies and RCTs.^3^ Conflicting evidence exists for blood pressure and T2D from previous MR studies.^14–18^ Previous MR studies for IHD and BMI did not support causal associations.^19–22^ Although associations between vitamin D and the risk of depression and non-vertebral fracture were suggestive from observational studies and RCTs, our MR analysis found no evidence of causality for their associations. These finding are further supported by the recently published MR studies,^23^^,^^24^ in which the effects of vitamin D on major depression (59 851 cases)^23^ and fracture (185 057 cases)^24^ are investigated, respectively, but none of them supports any causality. In agreement with MR findings, a recent systematic literature review and meta-analysis investigating the effect of calcium, vitamin D or a combination of calcium and vitamin D supplements on the incidence of fractures (33 RCTs included, involving 51 145 participants) did not find any association between vitamin D or calcium plus vitamin D supplements and the incidence of non-vertebral fractures.^25^ There was no evidence on the association between vitamin D and all-cause mortality from previous observational studies and RCTs, although a previous MR study reported a significant effect [10 349 deaths, 95 766 total participants, OR = 1.30, 95% confidence interval (CI): 1.05 to 1.61].^26^ This study employed four SNPs in two loci (*DHCR7* and *CYP2R1*) as their IV, which explained only 1.0% of the variance for the 25(OH)D level. However, in our study with 85% power for detecting an effect of 1.2 and with a comparable case size (*N* = 9830 deaths), we did not observe a causal effect (OR = 1.030, 95% CI: 0.869 to 1.222, *P *=* *0.671). The association between vitamin D and all-cause mortality needs to be studied by a larger MR or meta-analysis of MR studies.

### Strengths and limitations

Our study has several strengths. This is the first study to investigate the causal effect of vitamin D in a large sample of 339 256 individuals across the whole spectrum of disease outcomes. Taking advantage of the PheWAS design, we tested the association between the 25(OH)D genetic-risk score and a wide spectrum of phenotypes. Then, we applied multiple MR methods, including two-stage MR, IVW MR and Egger’s MR, to explore the causal effect and test the robustness of our findings across multiple methods.

The study also has some limitations. In our analysis, we only included White individuals residing at a high latitude, which may hinder the generalizability of our findings to other populations. The weights we employed in the score creation were from a meta-analysis of GWAS,^9^ which covered individuals residing in multiple countries. Therefore, the distribution of vitamin D levels in the UK population may be different from that in the wider White population and thus the actual coefficients for variants might also differ. Another implication of the high latitude is that, in the UK Biobank population, an overall low 25(OH)D level (independent of genetic variation but due to a lack of adequate sunlight exposure) may increase the risk of vitamin D-related disease. Therefore, small changes in 25(OH)D levels due to genetic variation may not affect the disease risks further. Furthermore, the variance of 25(OH)D explained by the six SNPs was 2.84% from the SUNLIGHT GWAS and 1.61% from the SOCCS controls. Although the *F*-statistics indicates a robust instrument, the variance explained is low. Moreover, considering participants in the UK Biobank are not representative of the UK population and there is evidence of a ‘healthy volunteer’ selection bias,^27^ it is possible that the healthy volunteer selection bias observed may contribute to the null findings in this study.

Furthermore, the relationship between the 25(OH)D level and the risk of diseases may be nonlinear. As shown by previous studies, vitamin D supplementation only shows treatment effects among individuals with baseline 25(OH)D levels of no more than 30 nmol/L. When all participants were analysed irrespective of their baseline 25(OH)D levels, there was no treatment effect.^28^^,^^29^ Thus, the effect of 25(OH)D on health outcomes may differ by baseline serum 25(OH)D level. Considering the potential divergent 25(OH)D levels of the UK population, it is possible that we missed the true association between 25(OH)D levels and diseases among individuals of certain 25(OH)D levels.

Although we incorporated EMR data and self-reported medical conditions in our definition of phenotypes, problems with reporting bias could have occurred in outcome definition. Incorporation of more data, including general-practice data, outpatient data, prescription data and even imaging data, would help improve the validity of case definition in PheWAS studies. We set the minimum case number per phenotype based on a simulation analysis of PheWAS power estimates.^10^ We therefore restricted the PheWAS analysis to outcomes with >200 cases. From PheWAS analysis, we noted that the association between the genetic score of the 25(OH)D levels and vitamin D deficiency was not significant. This is probably due to the limited statistical power. In particular, only 291 cases with vitamin D deficiency were identified in this cohort of 339 256 individuals, which is much fewer than would be expected if 25(OH)D levels were systematically tested. Additionally, for the MR analyses, we explored only nine outcomes with more than 80% power. There were some outcomes that had been shown to be associated with vitamin D in previous MR studies, including total adiponectin,^30^ multiple sclerosis,^31^ Alzheimer’s disease,^32^ cancer mortality,^26^ mortality excluding cancer and cardiovascular events,^26^ ovarian cancer,^33^ HDL-cholesterol,^16^ triglycerides^16^ and cognitive functions,^34^ but, due to limited statistical power or data availability, we did not include them in our final MR analysis.

## Conclusion

Our study suggested that there was no evidence of a large to moderate (OR >1.2) causal effect of vitamin D on a number of health outcomes, particularly for SBP, DBP, the risk of hypertension, T2D, IHD, BMI, depression, non-vertebral fracture and all-cause mortality. Further, larger studies, probably involving the joint analysis of data from several large biobanks, may be needed to investigate smaller causal effects that nevertheless could be important for public health due to the high prevalence of low vitamin D levels in many populations.

## Funding

This work was supported by Cancer Research UK (grant number C348/A18927) to M.D., E.T., H.C., S.F.; Cancer Research UK Career Development fellowship (grand number C31250/A22804) to E.T.; and the National Institutes of Health (grant number R01 HL133786) to W.Q.W.; X.M., X.L., Y.H. and H.W. were supported by the China Scholarship Council.

**Conflict of interest:** None declared.

## Supplementary Material

dyz182_Supplementary_Data
