# Supplementary material for: Phenome-wide Mendelian-randomization study of genetically determined vitamin D on multiple health outcomes using the UK Biobank study
Source: Int J Epidemiol. 2019 Sep 13;48(5):1425–34. doi: 10.1093/ije/dyz182 (PMC6857754; doi:10.1093/ije/dyz182)
Supplement: dyz182_Supplementary_Data [file dyz182_supplementary_data.docx]

Supplementary Material

**List of Contents**

Supplementary Methods 2

1. Mendelian Randomization Analysis 2

2. Conventional Phenome Wide Association Study analysis 2

3. Bayesian analysis framework analysis, TreeWAS 3

4. UK Biobank 3

5. Study of Colorectal Cancer in Scotland (SOCCS) 4

Supplementary Results 5

1. Results of the systematic literature review on vitamin D Mendelian Randomization studies 5

2. Generation of outcome groups using phecode 6

Supplementary Tables and Figures 7

S1 Table. Keywords and search strategy used in the systematic literature review for Mendelian Randomization studies on Vitamin D. 7

S2 Table. Findings from most powered or recent Mendelian Studies on each outcome. 8

S3 Table. Characteristics of baseline confounders. 12

S4 Table. PheWAS results (with adjustment for BMI) for all phenotypes with P value less than 0.05 14

S4 Table. PheWAS results (without adjustment for BMI) for all phenotypes with P value less than 0.05 17

S6 Table. Results for intercept items of Egger’s regressions. 20

S1 Figure. Flowchart for vitamin D MR systematic literature review 21

S2 Figure. Manipulation process of electronic medical records. 22

References 24

# Supplementary Methods

## 1. Mendelian Randomization Analysis

The idea of IV analysis was first introduced into the econometrics literature by Wright and later adopted into the statistical measurement error and causal inference literature.^1, 2^ There are three assumptions that must hold for a SNP to be a valid IV: 1) the SNP is associated with the exposure (here serum 25(OHD)); 2) the SNP is independent of the outcome conditional on the exposure and any confounders (no pleiotropy); 3) the SNP is independent of confounders.^3^ Genetic markers are good IVs since they are randomly allocated at inception and thus randomly distributed (Mendel’s second law). Therefore, genetic variants are unrelated to confounders at population level and associations between genotypes and diseases are protected from reverse causality.^4^

In our MR analysis, we firstly conducted a two-stage MR. Since the score we used in PheWAS is weighted by effect estimates from previous GWAS,^5^ it equalled the 25(OH)D level predicted by genetic variants. The PheWAS we implemented, which regressed phenotypes on the score, was equivalent to the first stage in two-stage MR. Therefore, we just took the coefficients for the score item from PheWAS, and the exponential of the coefficients was the MR estimate of the causal effect of 25(OH)D on outcome. We then conducted IVW MR and Egger’s MR to check the consistency of our results between different MR methods and account for any unbalanced pleiotropy of individual SNPs. Causal effect estimates from separate SNPs as instrumental variables can be pooled together using the IVW method. In brief, individual effect estimates are first calculated by the ratio method for every SNP, where the coefficient from the regression of outcome on individual SNP is divided by the effect estimates of SNP on biomarker exposure. Then, individual effect estimates are pooled together by a random-effects meta-analysis.^6^ The overall causal effect from multiple IVs estimated by IVW equals the coefficient from a weighted regression of IV-outcome association on IV-exposure association with the intercept constrained at zero. However, this method assumes that IVs are independent of the outcome conditional on exposure and confounders (i.e., the effect of all the IVs on the outcome is through the exposure), which is also called “exclusion restriction”. Pleiotropic effects of IVs would violate this assumption. Egger’s MR employs a less strict version of the exclusion restriction, the InSIDE condition, where the correlation between the genetic effects on the exposure and the direct effects of the genetic variants on the outcome is zero.^7^ By viewing a MR with multiple IVs as analogous to a meta-analysis, the bias caused by pleiotropy is analogous to small study bias in meta-analysis. Under the InSIDE assumption, applying a regression method for all the individual IVs without constraining the intercept to be zero, Egger’s MR tests whether there is unbalanced pleiotropy. Rejection of the null hypothesis of the intercept value equal to zero implies unbalanced pleiotropy, suggesting that some IVs have pleiotropic effects and these do not cancel out.^7^

## 2. Conventional Phenome Wide Association Study analysis

A major challenge of a PheWAS is the methodology of defining the phenotypes. This is commonly addressed by curated phenotyping based on the International Classification of Diseases (ICD) codes. One such system is the “phecode system”, developed in 2010 by Denny J *et al**.*^8^ In this system, ICD codes that represent a common aetiology are combined into the same phecode group, while clinically distinct phenotypes which are represented by a single code are put into separate phecode groups. In addition, there are codes that are unlikely to be informative in context of a PheWAS study (e.g., E codes in ICD9 which represent external causes of injury) and these codes are marked as ‘ignorable’ and not analysed as disease outcomes in PheWAS.^8^ Finally, controls are considered to be all study participants that do not belong to the examined or closely related “phecode(s)”.

In data preparation for the PheWAS, the hospital inpatient, cancer registry, and death registry data were first pooled together. Then for each participant, every distinctive code he/she ever had was aggregated and the number of episodes reporting on that specific code was counted. We then applied the phecode algorithm ^8^ to define the case/control status of every participant for every phenotype group in the phecode system. For instance, an individual with a record of the ICD10 code M05.2 (rheumatoid vasculitis) was coded as a case for the phecode group 714.1 (rheumatoid arthritis). In addition, this individual was coded as a case for the phecode groups 714 (inflammatory polyarthropathies), 709.7 (Unspecified diffuse connective tissue disease), 716.9 (Arthropathy, not otherwise specified), and 446.3 (Hypersensitivity angiitis). Furthermore, this individual was flagged as an exclusion for phecodes 714.2-716.0 (other autoimmune arthritis; these are highly related codes and cases for 714.1 and 714 are not appropriate controls), and 696-696.99 (psoriasis; these codes are also related with immune system). Finally, this individual was coded as control for all other phecode groups.^8^

## 3. Bayesian analysis framework analysis, TreeWAS

A drawback of the conventional PheWAS by phecode is that the phenotypes are in a tree structure (e.g., viral hepatitis as parent node, with viral hepatitis A, viral hepatitis B, viral hepatitis C and chronic hepatitis as child nodes), and thus correlated. Testing such a tree-structured phenotype spectrum with a general linear model, which assumes independence, results in over correction of the *P* value (when adjusting for multiple testing). This issue was addressed in a recently proposed Bayesian analysis framework, named TreeWAS.^9^ In a TreeWAS, phenotypes are organized into a tree structure following their definition (e.g., the tree of ICD10 codes). To model the structure across the whole tree, coefficients are allowed to evolve down the tree based on a Markov chain process. In short, two parameters define the transition probabilities controlling the Markov process, θ and π_1_. Parameter θ represents the expected correlation between phenotypes, and the coefficient at a parent node can be inherited by a child node with probability of e^-θ^, or can mutate to a new independent value with probability of 1- e^-θ^. This new independent value will be zero with a probability of 1- π_1_ or nonzero with a probability of π_1._ The marginal posterior probability (PP) at each node, which is the probability that the true coefficient for the node is nonzero, and the magnitude of the true effect using the maximum a posteriori (MAP) estimator can be estimated.^9^ This new approach is shown to increase statistical power by up to 20%, and can detect new hits which cannot be detected by conventional PheWAS.^9^

## 4. UK Biobank

UK Biobank participants donated blood samples and were genotyped with 2 similar customized arrays of 95% shared content, Affymetrix UK BiLEVE Axiom (~50,000 individuals) and Affymetrix UK Biobank Axiom (~450,000 participants). UK Biobank genotype imputation was conducted based on a merged reference panel of the Haplotype Reference Consortium (HRC) panel, the UK10K panel and the 1000 Genome Phase 3 panel.^10^ All of 6 SNPs used in current study were on the HRC panel.

In the current study, we used the UK Biobank genetic data which was released in July 2017 including array and imputation variants from 488 378 participants. Genetic quality control was done centrally by UK Biobank at Wellcome Trust Center for Human Genetics (WTCHG), University of Oxford, UK.^10^ Heterozygosity outliers, missing rate outliers, sex mismatches and any individual of sex chromosome aneuploidy were excluded from our analyses. We also restricted analyses to participants, who were self-reported as British and with a white ancestry (as determined by their genotyping information), and kept the largest number of unrelated individuals among participants who were induced to be 3^rd^ degree relatives or closer. After these restrictions 339 256 individuals were included in our final analysis.

A touchscreen questionnaire was completed by every participant at recruitment, with a variety of demographic, lifestyle and other phenotypes collected, including gender, date of birth, height, weight, time spent outdoors in summer/winter, average household income before tax, qualifications, alcohol intake frequency, participant’s home address and self-reported medical conditions. Outdoor activity time was reported as an integer (the number of hours participants spend outdoors in a typical summer/winter day). The home address of participants was represented by the east co-ordinate and north co-ordinate of the postcodes to which invitations were sent, using the Ordnance Survey reference (<http://biobank.ctsu.ox.ac.uk/crystal/docs/UKgrid.pdf>). Household income before tax, qualifications and alcohol intake were reported as categorical variables (**Table S3**). In addition, participants were also asked to report any current or previous medical conditions (e.g., vascular/heart problems diagnosed by doctor, diabetes diagnosed by doctor, cancer diagnosed by doctor, or other diseases diagnosed by doctor) (<http://biobank.ctsu.ox.ac.uk/crystal/refer.cgi?id=113241>).

UK Biobank participants were linked to their EMR data, including hospital inpatient, cancer registry and death registry data. The hospital inpatient data are sent to UK Biobank on an annual basis, and include data on admission and discharge, diagnostic and operation codes, maternity and psychiatric data. Records in National Health Service (NHS)-funded admitted patient care and private health care within NHS hospitals were included. The World Health Organization’s ICD codes are used to record diagnosis information. From 1997 onwards, episodes are coded in the ICD 10^th^ version. Any earlier records were coded using the ICD 9^th^ version. The inpatient data were constructed on an episode basis, and an individual could have several lines of records based on the number of episodes he/she ever had.

(<http://biobank.ctsu.ox.ac.uk/showcase/docs/HospitalEpisodeStatistics.pdf>). The cancer registry data are sent to UK Biobank by the Medical Research Information Service which is based on the NHS Information Centre (for participants residing at England and Wales) and Information Services Division, part of NHS Scotland (for participants residing at Scotland). The cancer registry data extracted comprised date of cancer diagnosis, age at cancer diagnosis, type of cancer (coded by ICD codes), reported occurrences of cancer, histology code and behaviour code (<http://biobank.ctsu.ox.ac.uk/crystal/docs/CancerLinkage.pdf>). Finally, death register data are also available in UK Biobank and include date of death, age at death, underlying cause of death (only one), contributory causes of death (could be multiple) and description of cause of death (<http://biobank.ctsu.ox.ac.uk/crystal/docs/DeathLinkage.pdf>).

## 5. Study of Colorectal Cancer in Scotland (SOCCS)

SOCCS is a prospective, population-based case-control study, aiming to reveal genetic and environmental factors that have an impact on colorectal cancer risk and survival outcome.^11^ The study was approved by the MultiCentre Research Ethics Committee for Scotland (reference number: 01/0/05) and by the Research and Development Office of NHS Lothian (reference number: 2003/W/GEN/05). Blood samples were taken and stored for genotyping and 25(OH)D measurement. Genotyping was conducted by OmniExpressExome BeadChip 8v1.1 or 8v1.250 (Illumina Inc., San Diego, CA).^11^ Details about genotyping methods and quality control was described elsewhere.^12^ Plasma 25(OH)D for each individual was measured by the liquid chromatography-tandem mass spectrometry (LC-MS/MS) method. In order to balance the effect of different seasons when blood samples were taken, a May-standardization algorithm was applied on the 25(OH)D level for each participant.^13^ A total of 2821 healthy controls from the SOCCS study were included in our study. Approximately 99% of them were self-reported European ancestry.

# Supplementary Results

## 1. Results of the systematic literature review on vitamin D Mendelian Randomization studies

There were 5 MR studies on glycaemic traits and diabetes,^14-18^ 5 MR studies on circulatory outcomes,^19-23^ 4 MR studies on autoimmune diseases,^24-27^ 3 MR studies on cognitive functions and related outcomes, ^28-30^ 3 MR studies on mortality or survival outcomes,^31-33^ 2 MR studies on cancer,^34, 35^ one MR study on each of the following outcomes: childhood caries,^36^ bone mineral density and bone metabolism biomarkers,^37^ birth weight,^38^ body mass index,^39^ paediatric asthma,^40^ C-reactive protein,^41^ and atherogenic lipoproteins.^42^ For a summary of the most recent or most powered study of each outcome, please see **Supplementary Table 2**.

Among the 5 studies on glycaemic traits and diabetes, two studies suggested significant associations.^15, 16^ The odds for risk of T2D was estimated to be 1.51 (95% CI: 0.98-2.33, *P*=0.04, determined by score of *DHCR7* (rs11234027 and rs7944926)), and 1.02 (95% CI: 0.75-1.37, *P*=0.84, determined by score of *CYP2R1* (rs10741657 and rs12794714)) per 20 nmol/L lower 25(OH)D level in three Danish cohorts of a total of 96 423 individuals (5037 T2D cases) by Afzal *et al*.^15^ As a risk factor for T2D, Husemoen et al. found that a doubling of 25(OH)D levels was associated with a 60.89% (95% CI: 17.51%-120.28%, *P*=0.003) higher total adiponectin.^16^ However, in a more recent and larger study, Ye et al. estimated the causal effect of vitamin D to be 1.01 (95% CI: 0.75-1.36, *P*=0.94) per 25.0 nmol/L (1 SD) lower 25(OH)D level for risk of T2D in a summary statistical analysis from multiple consortia of up to 104 488 individuals (28 144 cases).^18^

Among the 5 studies on circulatory outcomes, 3 studies were on blood pressure and risk of hypertension ^20 22 23^. Evidence from a previous MR study by Kunustor et al. did not find any significant association between vitamin D and SBP or DBP in a sample of 69 395 individuals.^20^ Similarly, the study by Skabby did not observe any significant association.^22^ However, in a MR study of summarized data, using summary results from multiple consortia (up to 50 studies), DBP and risk of hypertension were found to be significantly associated with vitamin D in a final sample of more than 140k (-0.29 mmHg (95% CI: -0.52~-0.07, *P* = 0.01) for DBP, OR = 0.92 (95% CI: 0.87~0.97, *P* = 0.002) for risk of hypertension for every 10% increase in 25(OH)D level).^23^ Brondum-Jacobsen et al. studied the effect of vitamin D on risk of ischemic heart disease (IHD) and myocardial infarction (MI) in a sample of 92 415 white Danish individuals (14 455 cases for IHD and 7061 cases for MI), but did not observe any significant association (for IHD, OR=0.98, 95% CI: 0.76-1.26, *P*=0.86; for MI: OR=1.15, 95% CI: 0.83-1.59, *P*=0.49 per 25 nmol/L lower 25(OH)D level).^19^ In a sample of 86 955 (22 233 cases) white individuals, Manousaki et al. did not find any effect of vitamin D on risk of coronary heart disease (CAD) (OR=0.99, 95% CI: 0.84-1.17, *P*=0.93 per SD decrease of log transformed 25(OH)D level).^21^

Two studies were on risk of multiple sclerosis (MS). In a white population of 38 589 (14 498 cases), the odds for MS was estimated to be 2.02 (95% CI: 1.65-2.46, *P*=7.72*10^-12^ per SD decrease of natural log transformed 25(OH)D level).^24^ In the second study, the effect was significant and consistent with the above study.^25^

For cognitive functions and related diseases, Mokry et al. investigated the effect on risk of Alzheimer’s disease (AD) in a white sample of 54 162 (17 008 cases). The OR was estimated to be 1.25 (95% CI: 1.03-1.51, *P*=0.021) per SD decrease of log transformed 25(OH)D level.^30^ For mortality and survival outcomes, Afzal et al. studied the association between vitamin D and mortality, including all-cause mortality, cardiovascular mortality, cancer mortality, and mortality due to all other causes in a population of 95 766. The effects of 25(OH)D level on all-cause mortality (OR=1.30, 95% CI: 1.05-1.61, per 20 nmol/L lower 25(OH)D level), cancer mortality (OR=1.43, 95% CI: 1.02-1.99, per 20 nmol/L lower 25(OH)D level) and other mortality (OR=1.44, 95% CI: 1.01-2.04, per 20 nmol/L lower 25(OH)D level) were found to be significant.^31^ In addition, Ong et al. studied the association between vitamin D and ovarian cancer in a population of 31 719 people. The OR for epithelial ovarian cancer (10 065 cases) was estimated to be 1.27 (95% CI: 1.06-1.51) per 20 nmol/L lower 25(OH)D level, and for high-grade serous ovarian cancer (4121 cases) it was estimated to be 1.54 (95% CI: 1.19-2.01) per 20 nmol/L lower 25(OH)D level.^34^ None of the other included MR studies found any statistically significant associations.

## 2. Generation of outcome groups using phecode

We downloaded the December 2016 version of the EMR data and there were 2 779 598 unique records of hospital inpatient data corresponding to 395 978 unique individuals (2 714 364 records had an ICD10 diagnosis code, 52 123 had an ICD9 diagnosis code and 13 111 records had no diagnosis code). In the cancer registry data there were 233 753 records corresponding to 79 066 unique participants (207 935 records had an ICD10 diagnosis code, and 25 818 had an ICD9 diagnosis code), and 14 417 records were included from the death registry data.

The phecode system for curating ICD-based phenotyping was applied in UK Biobank EMRs. A total of 88.6 % (3 106 440 out of 3 504 994) of records of unique ICD code per participant were successfully mapped to phecode (**Figure S2**). Among the unmatched records, 86.7% (345 426 out of 398 554 unmatched records) were ICD10 codes beginning with V (transport accidents) or Z (factors influencing health status and contact with health services), which are not expected to be directly associated with any genetic factor. In total 1853 disease outcomes were generated with a median number of cases of 309 (range: 0 to 160 512).

# Supplementary Tables and Figures

## Supplementary Table 1. Keywords and search strategy used in the systematic literature review for Mendelian Randomization studies on Vitamin D.

| **MEDLINE (OvidSP)**  1. Vitamin d/ or 25-OHD.mp. or 25 hydroxyvitamin D.mp. or cholecalciferol/  2. colecalciferol.mp. or hydroxycholecalciferols/ or hydroxycolecalciferols.mp.  3. calcifediol/ or dihydroxycholecalciferols/ or dihydroxycolecalciferols.mp.  4. calcitriol/ or 24,25-dihydroxyvitamin d 3/ or 24,25-OH2 D3.mp.  5. ergocalciferols/ or dihydrotachysterol/ or 25-hydroxyvitamin d 2/ or 25-OHD2.mp.  6. 1,25-dihydroxyvitamin d.mp. or 1,25-OH2 D.mp. or 1,25-dihydroxyvitamin d2.mp.  7. 1,25-dihydroxyergocalciferol.mp. or 1,25-OH2D2.mp. or 1,25-dihydroxyvitamin d3.mp. or 1,25-OH2 D3.mp. or ergocalciferols/  8. vitamin D2.mp. or vitamin D 2.mp. or vitamin D3.mp. or vitamin D 3.mp  9. 1 or 2 or 3 or 4 or 5 or 6 or 7 or 8  10. Mendelian Randomization Analysis/ or Mendelian randomisation.mp or Mendelian randomization.mp  11. instrumental variable.mp or instrumental variables.mp or genetic instrument.mp or genetic instruments.mp  12. random Mendelian.mp or genetic risk score.mp or genetic risk scores.mp or genetic score.mp or genetic scores.mp  13. 10 or 11 or 12  14. 9 and 13 |
| --- |
| **EMBASE (OvidSP)**  1. Vitamin d/ or 25-OHD.mp. or 25 hydroxyvitamin D/ or colecalciferol/ or cholecalciferol.mp.  2. hydroxycolecalciferols/ or hydroxycholecalciferols.mp.  3. calcifediol/ or dihydroxycolecalciferols/ or dihydroxycholecalciferols.mp.  4. calcitriol/ or secalciferol/ or 24,25-OH2 D3.mp. or ergocalciferol/ or dihydrotachysterol/  5. 25-hydroxyvitamin d 2.mp. or 25-OHD2.mp. or 1,25-dihydroxyvitamin d.mp. or 1,25-OH2 D.mp.  6. 1,25dihydroxyergocalciferol/ or 1,25-dihydroxyvitamin d2.mp. or 1,25-OH2 D2.mp.  7. 1,25-dihydroxyvitamin d3.mp. or 1,25-OH2 D3.mp. or ergocalciferol derivative/  8. vitamin D2.mp. or vitamin D 2.mp. or vitamin D3.mp. or vitamin D 3.mp  9. 1 or 2 or 3 or 4 or 5 or 6 or 7 or 8  10. Mendelian Randomization Analysis/ or Mendelian randomisation.mp or Mendelian randomization.mp  11. instrumental variable.mp or instrumental variables.mp or genetic instrument.mp or genetic instruments.mp  12. random Mendelian.mp or genetic risk score.mp or genetic risk scores.mp or genetic score.mp or genetic scores.mp  13. 10 or 11 or 12  14. 9 and 13 |

## Supplementary Table 2. Findings from most powered or recent Mendelian Studies on each outcome.

| **Study** | **Genetic Instruments** | **No/No of Events** | **Outcomes** | **Estimate of effect (95% CI)** | ***P* value** | **Unit of estimated effect** |
| --- | --- | --- | --- | --- | --- | --- |
| Ye Z. 2015 ^18^ | *CYP2R1*, *DHCR7*,  *DBP* and *CYP24A1* | 104 488/28 144 | T2D | 1.01 (0.75, 1.36) | 0.94 | 1 SD decrease in 25OHD level |
|  |  | 46 368 | Fasting glucoes | -0.02 (-0.04, 0.01) | 0.28 | mmol/L per SD decrease in 25OHD level |
|  |  | 46 368 | 2-h glucose | 0.08 (-0.06, 0.22) | 0.25 | mmol/L per SD decrease in 25OHD level |
|  |  | 46 368 | Fasting insulin | -1.04 (-3.91, 1.83) | 0.48 | % difference per SD decrease in 25OHD level |
|  |  | 46 368 | HbA1c | 0.01 (-0.04, 0.05) | 0.8 | % difference per SD decrease in 25OHD level |
| Vim K.S. 2014 ^23^ | *CYP2R1* and *DHCR7* | 146 581 | SBP | -0.37 (-0.73, 0.003) | 0.052 | mm Hg per 10% increase in 25OHD level |
|  |  | 142 255 | DBP | -0.29 (-0.52, -0.07) | 0.01 | mm Hg per 10% increase in 25OHD level |
|  |  | 142 255 | Risk of hypertension | 0.92 (0.87, 0.97) | 0.002 | per 10% increase in 25OHD level |
| Manousaki D. 2016 ^21^ | *DHCR7*, *CYP2R1*, *GC*, and *CYP24A1* | 86 995/22 233 | Coronary artery disease | 0.99 (0.84, 1.17) | 0.93 | 1 SD decrease in log-transfromed 25OHD level |
| Morkry L.E. 2015 ^24^ | *DHCR7*, *CYP2R1*, *GC*, and *CYP24A1* | 38 589/14 498 | Multiple sclerosis | 2.02 (1.65, 2.46) | 7.72E-12 | 1 SD decrease in log-transfromed 25OHD level |
| Morkry L.E. 2015 ^30^ | *DHCR7*, *CYP2R1*, *GC*, and *CYP24A1* | 54 162/17 008 | Alzheimer disease | 1.25 (1.03, 1.51) | 0.021 | 1 SD decrease in log-transfromed 25OHD level |
| Afzal S. 2014 ^31^ | *DHCR7* and *CYP2R1* | 95 766/10 349 | All-cause mortality | 1.3 (1.05, 1.61) | NA | 20 nmol/L lower 25OHD |
|  |  | 95 766/3231 | Cardiovascular mortality | 0.77 (0.55, 1.08) | NA | 20 nmol/L lower 25OHD |
|  |  | 95 766/2839 | Cancer mortality | 1.43 (1.02, 1.99) | NA | 20 nmol/L lower 25OHD |
|  |  | 95 766/2585 | Other mortality | 1.44 (1.01, 2.04) | NA | 20 nmol/L lower 25OHD |
| Ong J.S. 2016 ^34^ | *DHCR7*, *CYP2R1* and *GC* | 31 719/10 065 | All ovarian cancer | 1.27 (1.06, 1.51) | NA | 20 nmol/L lower 25OHD |
|  |  | 31 719/4121 | Serous subtype of ovarian cancer | 1.54 (1.19, 2.01) | NA | 20 nmol/L lower 25OHD |
| Theodoratou E. 2012 ^35^ | *DHCR7*, *CYP2R1*, *GC*, and *CYP24A1* | 4238/2001 | Colorectal cancer | 1.16 (0.60, 2.23) | NA | per unit increase in log 25OHD level |
| Dudding T. 2015 ^36^ | *DHCR7*, *CYP2R1* and *GC* | 5545 | Caries experience | 0.93 (0.83, 1.05) | 0.26 | per 10 nmol/L increase 25OHD level |
|  |  | 1933 | Early caries onset | 1.09 (0.89, 1.34) | 0.37 | per 10 nmol/L increase 25OHD level |
| Li S.S. 2016 ^37,a^ | *DHCR7*, *CYP2R1*, *GC*, and *CYP24A1* | 1824 | Lumar 1-4 BMD | -0.048 (-0.158, 0.062) | 0.384 | g/cm^2^ per unit increase in log-transformed 25OHD |
|  |  | 1824 | Femoral neck BMD | -0.044 (-0.120, 0.032) | 0.261 | g/cm^2^ per unit increase in log-transformed 25OHD |
|  |  | 1824 | Total hip BMD | -0.041 (-0.123, 0.041) | 0.326 | g/cm^2^ per unit increase in log-transformed 25OHD |
|  |  | 1824 | PTH | 0.088 (-0.034, 0.210) | 0.152 | pg/mL per unit increase in log-transformed 25OHD |
|  |  | 1824 | P1NP | -0.099 (-0.291, 0.093) | 0.312 | g/L per unit increase in log-transformed 25OHD |
| Tyrrell J. 2016 ^38^ | *DHCR7* and *CYP2R1* | 30 340 | Birth weight | -26 (-54, 2) | 0.13 | g per 10% lower 25OHD level |
| Vimaleswaran K.S. 2013 ^39^ | DHCR7 and CYP2R1 | 123 864 | BMI | -0.002 (-0.009, 0.005) | 0.57 | per risk allele |
|  | GC and CYP24A1 | 123 864 | BMI | 0.002 (-0.006, 0.009) | 0.67 | per risk allele |
| Hysinger E.B. 2016 ^40^ | *CYP2R1* and *GC* | 5080/1203 | Paediatric asthma | -0.0000351 (NA, NA) | 0.85 | NA |
|  |  | NA | Severe asthma exacerbations | -0.00833 (NA, NA) | 0.86 | NA |
| Liefaard M.C. 2015 ^41^ | *DHCR7*, *CYP2R1*, *GC*, and *CYP24A1* | 10 788 | CRP | -0.018 (NA, NA) | 0.082 | 1 SD change in 25OHD level |
| Ooi E.M. 2014 ^42^ | DHCR7 and CYP2R1 | 79 743 | Remnant cholesterol | 4.0 (-2.4, 11) | 0.22 | % per 50% decrease in 25OHD level |
|  |  | 79 812 | LDL cholesterol | 2.2 (-1.7, 6.2) | 0.28 | % per 50% decrease in 25OHD level |
|  |  | 85 363 | HDL cholesterol | -6.0 (-10, -2.3) | 0.001 | % per 50% decrease in 25OHD level |
| Dudding T 2018 ^43^ | GC, CYP2R1, DHCR7, CYP24A1, PDE3B | 5 133/5 984 | Oral cancer | 0.86 (0.68, 1.09) | 0.22 | per standard deviation increase in log transformed 25OHD |
| Chandler PD 2018 ^44^ | DHCR7, CYP2R1, GC | 3 985/23 294 women | Incident total cancer | 1.10 (0.96, 1.25) | 0.17 | 20 nmol/L higher 25OHD level as determined by genetic variants |
|  |  | 1 560 | Incident breast cancer | 1.14 (0.92, 1.41) | 0.22 | NA |
|  |  | 329 | Incident colorectal cancer | 1.54 (0.96, 2.46) | 0.07 | NA |
|  |  | 330 | Incident lung cancer | 0.96 (0.55, 1.68) | 0.89 | NA |
|  |  | 770 | Total cancer death | 0.98 (0.73, 1.32) | 0.9 | NA |
| Chen C 2019 ^45^ | DHCR7, CYP2R1, GC, CYP24A1 | 4 254 men | Total testosterone | 0.12 (0.02, 0.22) | NA | 1 SD increment of 25(OH)D determined by genetic variants |
| Larsson SC 2018 ^46^ | DHCR7, CYP2R1, GC, CYP24A1 | 32 965 | Femoral neck BMD | 0.02 (-0.03, 0.07) | 0.37 | 1 SD increment of 25(OH)D determined by genetic variants |
|  |  | 32 965 | Lumbar spine BMD | 0.02 (-0.04, 0.08) | 0.49 | 1 SD increment of 25(OH)D determined by genetic variants |
|  |  | 142 487 | Estimated BMD | -0.03 (-0.05, -0.01) | 0.02 | 1 SD increment of 25(OH)D determined by genetic variants |
| Sun JY 2019 ^47^ | DHCR7, CYP2R1, GC, CYP24A1, SEC23A, AMDHD1 | 66 628 | Total boday BMD | 0.92 (0.82, 1.04) | 0.17 | NA |
| Dimitrakopoulou VI 2017 ^48^ | DHCR7, CYP2R1, GC, CYP24A1 | 11 488 | Colorectal cancer | 0.92 (0.76, 1.10) | 0.36 | per 25 nmol/L increase in genetically determined 25OHD level |
|  |  | 15 748 | Breast cancer | 1.05 (0.89, 1.24) | 0.59 | per 25 nmol/L increase in genetically determined 25OHD level |
|  |  | 22 898 | Prostate cancer | 0.89 (0.77, 1.02) | 0.08 | per 25 nmol/L increase in genetically determined 25OHD level |
|  |  | 4 369 | Ovarian cancer | 1.12 (0.86, 1.47) | 0.4 | per 25 nmol/L increase in genetically determined 25OHD level |
|  |  | 12 537 | Lung cancer | 1.03 (0.87, 1.23) | 0.72 | per 25 nmol/L increase in genetically determined 25OHD level |
|  |  | 1 896 | Pancreatic cancer | 1.36 (0.81, 2.27) | 0.25 | per 25 nmol/L increase in genetically determined 25OHD level |
|  |  | 1 627 | Neuroblastoma cancer | 0.76 (0.47, 1.21) | 0.24 | per 25 nmol/L increase in genetically determined 25OHD level |
| Sun Y.Q. 2018 ^49^ | GC,DHCR7 and CYP2R1 | 54 580 /676 | Lung cancer | 0.96 (0.54,1.69) | 0.88 | 25 nmol/L increase in 25OHD |
| Ong J. S. 2018 ^50^ | GC, CYP2R1, DHCR7, and CYP24A1 | 438 870/46 155 | Cancer | 0.97 (0.91,1.04) | 0.4 | 20 nmol/L increase in 25OHD |
|  |  | 438 870/6 998 | Cancer mortality | 0.97 (0.84,1.11) | 0.54 | 20 nmol/L increase in 25OHD |
| Aspelund T 2019 ^51^ | DHCR7, CYP2R1 | 10 501/4 003 | All-cause mortality | 1.32 (0.80, 2.24) | NA | per 20 nmol/L decrease in genetically determined 25OHD level |
|  |  | 10 501/4 003 | All-cause mortality | 1.35 (0.81, 2.37) | NA | NA |
| Gianfrancesco MA 2017 ^52^ | GC, CYP2R1, DHCR7 | 16 820/569 | Paediatric onset multiple sclerosis | 0.72 (0.55, 0.94) | 0.02 | NA |
| He Y 2018 ^53^ | DHCR7, CYP2R1, GC, CYP24A1, SEC23A, AMDHD1 | 48 168/18 967 | Colorectal cancer | 0.91 (0.69, 1.19) | 0.475 | per unit log transformed 25OHD change determiend by genetic variants |
| Cuellar-Partida G. 2017 ^54^ | DHCR7, CYP2R1, GC, CYP24A1 | 37 382 European | Myopic refractive error | -0.02 (-0.09, 0.04) | NA | per 10 nmol/L increase in 25OHD level |
|  |  | 8 376 Asian | Myopic refractive error | 0.01 (-0.17, 0.19) | NA | per 10 nmol/L increase in 25OHD level |
| Winslow UC 2018 ^55^ | DHCR7, CYP2R1 | 1 569/103 084 | Non-melanoma skin cancer | 1.11 (0.91, 1.35) | NA | 20 nmol/L higher 25OHD level as determined by genetic variants |
| Havdahl A 2019 ^56^ | DHCR7, CYP2R1, GC, CYP24A1, SEC23A, AMDHD1 | 19 526/327 478 | Fatigue | 1.05 (0.87, 1.27) | 0.62 | per 1sd decrease log transformed 25OHD level |
| Takahashi H 2018 ^57^ | DHCR7, CYP2R1, GC, CYP24A1 | 12 488/18 169 | Glioma | 1.21 (0.90, 1.62) | 0.201 | NA |
| Larsson SC 2017 ^58^ | DHCR7, CYP2R1, GC, CYP24A1 | 17 008/37 154 | Alzheimer's disease | 0.92 (0.85, 0.98) | 0.01 | per 20% higher levels |
| Teumer A 2018 ^59^ | DHCR7, CYP2R1, GC | 133 720 | Estimated glomerular filtration rate | -0.013 | 0.003 | NA |
|  |  | 54 448 | Urinary albumin: creatinine ratio | 0.032 | 0.265 | NA |
| Manousaki D. 2017 ^60^ | GC, CYP2R1, DHCR7, and CYP24A1 | 146 761/25 109 | Asthma | 1.03 (0.90,1.19) | 0.63 | 1 SD decrease in log-transfromed 25OHD level |
|  |  | 15 008/7 047 | Childhood onset asthma | 0.95 (0.69,1.31) | 0.76 | 1 SD decrease in log-transfromed 25OHD level |
|  |  | 40 835/10 788 | Atopic dermatitis | 1.12 (0.92,1.37) | 0.27 | 1 SD decrease in log-transfromed 25OHD level |
|  |  | 12 853 | Elevated IgE level | −0.40 ( −1.65,0.85) | 0.54 | 1 SD decrease in log-transfromed 25OHD level |
| Bowman K.2019 ^61^ | GC,CYP2R1, DHCR7, CYP24A1, SEC23A, and AMDHD1 | 313 121/544 | Delirium | 0.74 (0.62,0.87) | 0.0004 | 10 nmol/L increase in 25OHD level |
| Bae S.C.2018 ^62^ | SSTR4, GC, and NADSYN1 | 4 744/2 104 | Systemic lupus erythematosus | 0.032 (-0.201,0.265) | 0.789 | NA |
|  |  | 41 282/12 307 | Rheumatoid arthritis | 0.026 (-0.094,0.146) | 0.664 | NA |
| Magnus M. C. 2018 ^63^ | GC, CYP2R1, DHCR7, and CYP24A1 | 9 447 | Gestational hypertension | 0.90 (0.78,1.03) | NA | 10% decrease in 25OHD |
|  |  | 9 447 | Preeclampsia | 0.98 (0.89,1.07) | NA | 10% decrease in 25OHD |
| Wang N. 2018 ^64^ | GC, CYP2R1, DHCR7, and CYP24A1 | 9 182 | Non-alcoholic fatty liver disease | 1.03 (0.99, 1.07) | NA | NA |
| Lund-Nielsen J. 2018 ^65^ | CYP2R1, DHCR7, CYP24A1, SEC23A, and AMDHD1 | 115 110/653 | Crohn disease | 0.98 (0.94 ,1.03) | NA | 1.4-nmol/L increase in 25OHD |
|  |  | 115 110/1 265 | Ulcerative colitis | 1.01 (0.97,1.05) | NA | 1.4-nmol/L increase in 25OHD |
| Larsson S. 2018 ^66^ | CYP2R1, DHCR7, CYP24A1, SEC23A, and AMDHD1 | 54 162/17 008 | Alzheimer's Disease | 0.86 (0.78,0.94) | 0.002 | 1 SD increase in 25OHD level |
| Michaelsson K. 2018 ^67^ | GC,CYP2R1, DHCR7, CYP24A1, SEC23A, and AMDHD1 | 173 005/59 851 | Major depression | 1.02 (0.97,1.08) | 0.44 | 1 SD decrease in 25OHD level |
| Larsson S. 2018 ^68^ | GC,CYP2R1, DHCR7, CYP24A1, SEC23A, and AMDHD1 | 438 847/34 217 | Ischemic stroke | 1.01 (0.94,1.08) | 0.84 | 1 SD increase in 25OHD level |
| Tan V. Y. 2018 ^69^ | GC,CYP2R1, DHCR7, CYP24A1, SEC23A, and AMDHD1 | 72 729 | Circulating insulin-like growth factor binding protein 3 (IGFBP-3) | 0.11 (−0.10,0.31) | 0.32 | 1 unit increase in log-transformed 25OHD level |
| Mai X.M. 2019 ^70^ | GC, CYP2R1, DHCR7, and CYP24A1 | 56 435 | High-density lipoprotein | 2.52 (0.79,4.25) | 0.004 | 25 nmol/L increase in 25(OH)D |
|  |  | 56 435 | Total cholesterol | 0.60 (- 0.73,1.94) | 0.38 | 25 nmol/L increase in 25(OH)D |
|  |  | 56 435 | Non-HDL cholesterol | - 2.74 ( - 6.16, 0.67) | 0.96 | 25 nmol/L increase in 25(OH)D |
| Larsson S.C. 2017 ^71^ | GC, CYP2R1, DHCR7, and CYP24A1 | 17 352/5 333 | Parkinson's disease | 0.98 (0.93,1.04) | 0.56 | 10% decrease in 25OHD |
| Noordam R. 2017 ^72^ | GC,NADSYN1, and CYP2R1 | 4 492 | Perceived age | 0.030 (-0.015,0.075) | 0.18 | 1 unit increase in genetic risk score |
|  |  | 4 492 | Degree of skin wrinkling | 0.000 (-0.054,0.054) | 1 | 1 unit increase in genetic risk score |
|  |  | 4 492 | Degree of pigmented spots | 0.055 (-0.004,0.114) | 0.07 | 1 unit increase in genetic risk score |
| Dong J. 2019 ^73^ | GC,CYP2R1, DHCR7, CYP24A1, SEC23A, and AMDHD1 | 23 326/6 167 | Esophageal adenocarcinoma | 0.68 (0.39,1.19) | 0.18 | 20 nmol/L increase in 25OHD |
|  |  | 23 326/4 112 | Barrett's esophagus | 1.21 (0.77,1.92) | 0.41 | 20 nmol/L increase in 25OHD |
| Lu L 2018 ^74^ | DHCR7, CYP2R1 | 58 312/370 592 | Diabetes | 0.86 (0.77, 0.97) | 0.01 | 25 nmol/L high 25OHD as determined by genetic variants |
|  | DHCR7, CYP2R1, GC, CYP24A1 | 32 796/248 629 | Diabetes | 0.92 (0.84, 1.01) | 0.07 | 25 nmol/L high 25OHD as determined by genetic variants |
| Trajanoska K. 2018 (REF) | DHCR7, CYP2R1, GC, CYP24A1 | 562258/185057 | Fracture risk | 0.84 （0.70， 1.02） | 0.07 | per SD decrease of genetically determined 25OHD level |

^a^ study in Chinese population, all other studies were in white population.

For each outcome, the study with the largest sample size was reported in this table. If there were two studies of similar sizes, the most recent one is reported.

## Supplementary Table 3. Characteristics of baseline confounders.

| Variable Name | Feature |  |
| --- | --- | --- |
| *Continuous* | Mean (S.D.) | Number of missingness (%) |
| Age | 56.89 (7.99) years | 0 |
| Standing height | 168.80 (9.24) cm | 730 (0.22%) |
| Weight | 78.31 (15.88) kg | 964 (0.28%) |
| BMI | 27.40(4.76) kg/m^2^ | 1084 (0.32%) |
| Time spend outdoors in summer | 3.17 (3.58) hours/day | 319 (0.09%) |
| Time spend outdoors in winter | 0.14 (4.71) hours/day | 319 (0.09%) |
| *Categorical* | Levels | Number of participants (%) |
| Sex | Male | 157 146 (46.32%) |
|  | Female | 182 110 (53.68%) |
|  | Missingness | 0 |
| Household income  before tax | Less than £18 000 | 63 806 (18.81%) |
|  | £18 000 to £30 999 | 75 135 (22.15%) |
|  | £31 000 to £51 999 | 77 268 (22.78%) |
|  | £52 000 to £100 000 | 60 478 (17.83%) |
|  | Greater than £100 000 | 15 678 (4.62%) |
|  | Do not know | 13 027 (3.84%) |
|  | Prefer not to answer | 32 756 (9.66%) |
|  | Missingness | 1108 (0.33%) |
| Qualifications | College or University degree | 107 150 (31.58%) |
|  | A levels/AS levels or equivalent | 38 467 (11.34%) |
|  | O levels/GCSEs or equivalent | 74 674 (22.01%) |
|  | CSEs or equivalent | 18 231 (5.37%) |
|  | NVQ or HND or HNC or equivalent | 22 411 (6.61%) |
|  | Other professional qualifications  (eg: nursing, teaching) | 17 418 (5.13%) |
|  | None of the above | 57 809 (17.04%) |
|  | Prefer not to answer | 2777 (0.82%) |
|  | Missingness | 319 (0.09%) |
| Alcohol intake  frequency | Daily or almost daily | 72 786 (21.45%) |
|  | 3 or 4 times a week | 82 022 (24.18%) |
|  | 1 or 2 times a week | 89 054 (26.25%) |
|  | 1 to 3 times a month | 37 492 (11.05%) |
|  | Special occasions only | 35 601 (10.49%) |
|  | Never | 22 065 (6.50%) |
|  | Prefer not to answer | 236 (0.07%) |
|  | Missingness | 0 |
| Assessment centre | Barts | 5908 (1.74%) |
|  | Birmingham | 16 056 (4.73%) |
|  | Bristol | 30 830 (9.09%) |
|  | Bury | 20 383 (6.01%) |
|  | Cardiff | 12 781 (3.77%) |
|  | Croydon | 15 076 (4.44%) |
|  | Edinburgh | 12 355 (3.64%) |
|  | Glasgow | 12 693 (3.74%) |
|  | Hounslow | 14 763 (4.35%) |
|  | Leeds | 31 348 (9.24%) |
|  | Liverpool | 22 685 (6.69%) |
|  | Manchester | 9078 (2.68%) |
|  | Middlesborough | 15 295 (4.51%) |
|  | Newcastle | 26 285 (7.75%) |
|  | Nottingham | 24 411 (7.20%) |
|  | Oxford | 9922 (2.92%) |
|  | Reading | 21 249 (6.26%) |
|  | Sheffield | 21 885 (6.45%) |
|  | Stockport(pilot) | 319 (0.09%) |
|  | Stoke | 13 824 (4.07%) |
|  | Swansea | 1620 (0.48%) |
|  | Wrexham | 490 (0.14%) |
|  | Missingness | 0 |

BMI, body mass index, is calculated by weight (kilograms) divided by the square of standing height (metres).

## Supplementary Table 4. PheWAS results (with adjustment for BMI) for all phenotypes with *P* value less than 0.05.

| **Description** | **Phecode** | ***P* value** | **N total** | **N cases** |
| --- | --- | --- | --- | --- |
| Delirium | 290.2 | 1.83*10^-4^ | 330 260 | 517 |
| Nephrotic syndrome | 580.2 | 9.75*10^-4^ | 324 211 | 374 |
| Vitamin D deficiency | 261.4 | 0.00116 | 333 355 | 291 |
| Viral infection | 79 | 0.00120 | 333 694 | 3324 |
| Calculus of ureter | 594.3 | 0.00170 | 330 521 | 2001 |
| Pilonidal cyst | 686.3 | 0.00248 | 326 359 | 475 |
| Urinary calculus | 594 | 0.00414 | 333 986 | 5466 |
| Otitis externa | 380.1 | 0.00451 | 334 279 | 411 |
| Gastritis and duodenitis | 535 | 0.00483 | 324 065 | 13 782 |
| Stricture of artery | 447.1 | 0.00534 | 328 472 | 689 |
| Other disorders of peritoneum | 568 | 0.00588 | 274 127 | 3123 |
| Symptoms of the muscles | 772 | 0.00594 | 335 035 | 523 |
| Giant cell arteritis | 446.5 | 0.00677 | 328 114 | 331 |
| Diseases of the oral soft tissues, excluding lesions specific for gingiva and tongue | 528 | 0.00701 | 332 143 | 1664 |
| Cancer of larynx | 149.4 | 0.00844 | 333 353 | 260 |
| Open wound or laceration of eye or eyelid | 870.1 | 0.00976 | 328 470 | 215 |
| Seborrheic keratosis | 702.2 | 0.0101 | 333 102 | 2498 |
| Sebaceous cyst | 706.2 | 0.0112 | 334 411 | 7255 |
| Hyperhidrosis | 705.8 | 0.0146 | 327 621 | 465 |
| Ventral hernia | 550.5 | 0.0153 | 299 402 | 2846 |
| Visual field defects | 368.4 | 0.0158 | 332 692 | 266 |
| Acute pain | 338.1 | 0.0171 | 334 763 | 318 |
| Cancer of urinary organs (incl. kidney and bladder) | 189 | 0.0176 | 333 862 | 2453 |
| Peritoneal adhesions (postoperative) (postinfection) | 568.1 | 0.0190 | 273 537 | 2533 |
| Diseases of hair and hair follicles | 704 | 0.0190 | 333 858 | 4154 |
| Malignant neoplasm of other urinary organs | 189.4 | 0.0220 | 334 248 | 2839 |
| Anomalies of tooth position/malocclusion | 524.3 | 0.0221 | 322 200 | 260 |
| Traumatic amputation | 872 | 0.0248 | 328 639 | 384 |
| Calculus of kidney | 594.1 | 0.0250 | 331 134 | 2614 |
| Digestive congenital anomalies | 750 | 0.0263 | 333 833 | 591 |
| Large cell lymphoma | 202.24 | 0.0283 | 331 413 | 602 |
| Malignant neoplasm of other and ill-defined sites within the digestive organs and peritoneum | 159 | 0.0287 | 325 783 | 4282 |
| Pyogenic granuloma | 686.4 | 0.0306 | 326 175 | 291 |
| Hydronephrosis | 595 | 0.0319 | 330 107 | 1587 |
| Hemiplegia | 342 | 0.0326 | 303 170 | 1147 |
| Chronic liver disease and cirrhosis | 571 | 0.0342 | 327 722 | 468 |
| Other disorders of cervical region | 723 | 0.0345 | 313 027 | 329 |
| Appendiceal conditions | 540 | 0.0346 | 335 056 | 3109 |
| Hallux valgus (Bunion) | 735.3 | 0.0373 | 328 944 | 5337 |
| Peripheral vascular disease, unspecified | 443.9 | 0.0393 | 329 849 | 2066 |
| Chronic renal failure [CKD] | 585.3 | 0.0419 | 325 911 | 2074 |
| Intestinal infection due to C. difficile | 8.52 | 0.0425 | 328 391 | 526 |
| Inflammation of eyelids | 371.3 | 0.0433 | 329 213 | 1929 |
| Other derangement of joint | 742.9 | 0.0439 | 306 450 | 1630 |
| Congenital anomalies of female genital organs | 751.11 | 0.0453 | 333 605 | 363 |
| Pneumoconiosis | 500.2 | 0.0477 | 326 492 | 824 |
| Other upper respiratory disease | 479 | 0.0482 | 331 853 | 11 950 |
| Internal derangement of knee | 835 | 0.0486 | 333 363 | 12 567 |
| Spinal cord injury without evidence of spinal bone injury | 952 | 0.0492 | 334 845 | 339 |
| Nontoxic multinodular goiter | 241.2 | 0.0493 | 321 540 | 535 |
| Cellulitis and abscess of face/neck | 681.2 | 0.0494 | 326 305 | 421 |
| Abnormal function study of cardiovascular system | 429.2 | 0.0499 | 330 370 | 438 |

## Supplementary Table 5. PheWAS results (without adjustment for BMI) for all phenotypes with *P* value less than 0.05.

| **Description** | **Phecode** | ***P* value** | **N total** | **N cases** |
| --- | --- | --- | --- | --- |
| Delirium | 290.2 | 3.37*10^-4^ | 331256 | 531 |
| Nephrotic syndrome | 580.2 | 7.86*10^-4^ | 325171 | 386 |
| Viral infection | 79 | 0.00125 | 334760 | 3344 |
| Vitamin D deficiency | 261.4 | 0.00163 | 334412 | 294 |
| Calculus of ureter | 594.3 | 0.00232 | 331542 | 2011 |
| Pilonidal cyst | 686.3 | 0.00249 | 327313 | 476 |
| Gastritis and duodenitis | 535 | 0.00356 | 325103 | 13854 |
| Stricture of artery | 447.1 | 0.00430 | 329478 | 707 |
| Urinary calculus | 594 | 0.00531 | 335049 | 5518 |
| Giant cell arteritis | 446.5 | 0.00580 | 329103 | 332 |
| Otitis externa | 380.1 | 0.00618 | 335348 | 415 |
| Diseases of the oral soft tissues, excluding lesions specific for gingiva and tongue | 528 | 0.00630 | 333205 | 1671 |
| Open wound or laceration of eye or eyelid | 870.1 | 0.00721 | 329482 | 218 |
| Symptoms of the muscles | 772 | 0.00735 | 336104 | 533 |
| Other disorders of peritoneum | 568 | 0.00836 | 274886 | 3146 |
| Seborrheic keratosis | 702.2 | 0.00994 | 334170 | 2507 |
| Visual field defects | 368.4 | 0.0120 | 333749 | 270 |
| Sebaceous cyst | 706.2 | 0.0130 | 335474 | 7276 |
| Cancer of larynx | 149.4 | 0.0154 | 334421 | 264 |
| Hyperhidrosis | 705.8 | 0.0159 | 328668 | 470 |
| Traumatic amputation | 872 | 0.0159 | 329651 | 387 |
| Cancer of urinary organs (incl. kidney and bladder) | 189 | 0.0168 | 334926 | 2463 |
| Diseases of hair and hair follicles | 704 | 0.0182 | 334918 | 4171 |
| Ventral hernia | 550.5 | 0.0189 | 300330 | 2861 |
| Malignant neoplasm of other urinary organs | 189.4 | 0.0221 | 335315 | 2852 |
| Acute pain | 338.1 | 0.0233 | 335829 | 325 |
| Peripheral vascular disease, unspecified | 443.9 | 0.0248 | 330873 | 2102 |
| Peritoneal adhesions (postoperative) (postinfection) | 568.1 | 0.0261 | 274285 | 2545 |
| Anomalies of tooth position/malocclusion | 524.3 | 0.0271 | 323226 | 262 |
| Chronic renal failure [CKD] | 585.3 | 0.0273 | 326894 | 2109 |
| Hydronephrosis | 595 | 0.0278 | 331133 | 1602 |
| Appendiceal conditions | 540 | 0.0281 | 336128 | 3123 |
| Chronic liver disease and cirrhosis | 571 | 0.0295 | 328737 | 472 |
| Calculus of kidney | 594.1 | 0.0296 | 332168 | 2637 |
| Digestive congenital anomalies | 750 | 0.0297 | 334899 | 592 |
| Large cell lymphoma | 202.24 | 0.0326 | 332467 | 603 |
| Pyogenic granuloma | 686.4 | 0.0330 | 327130 | 293 |
| Inflammation of eyelids | 371.3 | 0.0357 | 330257 | 1934 |
| Other derangement of joint | 742.9 | 0.0368 | 307338 | 1635 |
| Pneumoconiosis | 500.2 | 0.0402 | 327471 | 854 |
| Cervical intraepithelial neoplasia [CIN] [Cervical dysplasia] | 180.3 | 0.0423 | 312039 | 5254 |
| Spinal cord injury without evidence of spinal bone injury | 952 | 0.0429 | 335914 | 345 |
| Hallux valgus (Bunion) | 735.3 | 0.0432 | 329928 | 5353 |
| Other upper respiratory disease | 479 | 0.0446 | 332912 | 11988 |
| Malignant neoplasm of other and ill-defined sites within the digestive organs and peritoneum | 159 | 0.0448 | 326816 | 4301 |
| Noninflammatory disorders of vagina | 619.4 | 0.0472 | 298281 | 1436 |
| Disease of capillaries | 448 | 0.0484 | 334978 | 6207 |
| Congenital anomalies of female genital organs | 751.11 | 0.0493 | 334672 | 365 |
| Internal derangement of knee | 835 | 0.0499 | 334422 | 12611 |

## Supplementary Table 6. Results for intercept items of Egger’s regressions.

| **Phenotype** | **beta** | **s.e.** | ***P* value** |
| --- | --- | --- | --- |
| SBP | -0.026 | 0.049 | 0.628 |
| DBP | -0.033 | 0.024 | 0.236 |
| hypertension | -0.008 | 0.008 | 0.366 |
| T2D | -0.017 | 0.011 | 0.198 |
| IHD | -0.003 | 0.010 | 0.748 |
| BMI | 0.013 | 0.010 | 0.260 |
| Depression | 0.019 | 0.012 | 0.203 |
| Non-vertebral fracture | 0.002 | 0.011 | 0.879 |
| All-cause mortality | -0.007 | 0.012 | 0.614 |

Beta, standard error (s.e.) and *P* values for intercept items of Egger’s regression for every outcome. Significance (*P*<0.05) of the intercept item indicates existence of pleiotropy.

Figure S1. Flowchart for vitamin D MR systematic literature review.

Publications identified from Medline and Embase

(n = 182, all went to full text review)

Excluded (n = 121):

Not Vitamin D MR (n = 50)

Review articles (n = 32)

Conference abstracts (n = 30)

Comments/editorial/thesis (n = 9)

61 included after full text review

2 articles added (tracking google scholar, PubMed and reference lists)

Publications included (n = 63)

## Figure S2. Manipulation process of electronic medical records.

**Primary diagnosis codes**

**ICD 9 = 52 123**

**ICD 10 = 2 714 364**

**Secondary diagnosis codes**

**ICD 9 = 20 178**

**ICD 10 = 4 939 760**

**Death registry codes**

**ICD 10 = 37 287**

**Cancer registry codes**

**ICD 9 = 25 818**

**ICD 10 = 207 935**

**Counted**

**By participant**

**Number of rows:**

**3 504 994**

**Pooled together**

**A total of 7 997 465 codes**

We assessed electronical medical records of participants in December 2016, and downloaded their primary diagnosis codes (the primary cause for admission in every hospitalisation episode, one for each episode), secondary diagnosis codes (other existing medical conditions, could be several for each episode), and participants’ cancer registry and death registry codes through linkage with cancer registry and death registry. Then we treated every presence of codes as a unique line and pooled the four sources of codes we downloaded together, and got a total of 7 997 465 rows of ICD codes. Subsequently, presences of codes were counted and aggregated by participants (e.g., in the previous table, 3 records of the same code for an individual were three different lines, however, after the counting, they were presented by a single line with a new column featuring the number of records of the code). We got a table of 3 504 994 rows. At last, this table was mapped to phecode with the phecode mapping file (<https://phewascatalog.org/phecodes)> ^8^.

## References

1. Greenland S. An introduction To instrumental variables for epidemiologists. *Int J Epidemiol* 2000;**29**(6):1102.

2. Thomas DC, Conti DV. Commentary: the concept of 'Mendelian Randomization'. *Int J Epidemiol* 2004;**33**(1):21-5.

3. VanderWeele TJ, Tchetgen Tchetgen EJ, Cornelis M, Kraft P. Methodological challenges in mendelian randomization. *Epidemiology* 2014;**25**(3):427-35.

4. Lawlor DA, Harbord RM, Sterne JA, Timpson N, Davey Smith G. Mendelian randomization: using genes as instruments for making causal inferences in epidemiology. *Stat Med* 2008;**27**(8):1133-63.

5. Jiang X, O'Reilly PF, Aschard H, et al. Genome-wide association study in 79,366 European-ancestry individuals informs the genetic architecture of 25-hydroxyvitamin D levels. *Nat Commun* 2018;**9**(1):260.

6. Burgess S, Butterworth A, Thompson SG. Mendelian randomization analysis with multiple genetic variants using summarized data. *Genet Epidemiol* 2013;**37**(7):658-65.

7. Bowden J, Davey Smith G, Burgess S. Mendelian randomization with invalid instruments: effect estimation and bias detection through Egger regression. *Int J Epidemiol* 2015;**44**(2):512-25.

8. Denny JC, Ritchie MD, Basford MA, et al. PheWAS: demonstrating the feasibility of a phenome-wide scan to discover gene-disease associations. *Bioinformatics* 2010;**26**(9):1205-10.

9. Cortes A, Dendrou CA, Motyer A, et al. Bayesian analysis of genetic association across tree-structured routine healthcare data in the UK Biobank. *Nat Genet* 2017;**49**(9):1311-18.

10. Bycroft C. Genome-wide genetic data on ~500,000 UK Biobank participants. *bioRxiv* 2017.

11. Timofeeva MN, Kinnersley B, Farrington SM, et al. Recurrent Coding Sequence Variation Explains Only A Small Fraction of the Genetic Architecture of Colorectal Cancer. *Sci Rep* 2015;**5**:16286.

12. Tenesa A, Farrington SM, Prendergast JG, et al. Genome-wide association scan identifies a colorectal cancer susceptibility locus on 11q23 and replicates risk loci at 8q24 and 18q21. *Nat Genet* 2008;**40**(5):631-7.

13. Zgaga L, Theodoratou E, Farrington SM, et al. Plasma vitamin D concentration influences survival outcome after a diagnosis of colorectal cancer. *J Clin Oncol* 2014;**32**(23):2430-9.

14. Abbasi A. Mendelian randomization studies of biomarkers and type 2 diabetes. *Endocr Connect* 2015;**4**(4):249-60.

15. Afzal S, Brondum-Jacobsen P, Bojesen SE, Nordestgaard BG. Vitamin D concentration, obesity, and risk of diabetes: a mendelian randomisation study. *Lancet Diabetes Endocrinol* 2014;**2**(4):298-306.

16. Husemoen LL, Skaaby T, Martinussen T, et al. Investigating the causal effect of vitamin D on serum adiponectin using a Mendelian randomization approach. *Eur J Clin Nutr* 2014;**68**(2):189-95.

17. Leong A, Rehman W, Dastani Z, et al. The causal effect of vitamin D binding protein (DBP) levels on calcemic and cardiometabolic diseases: a Mendelian randomization study. *PLoS Med* 2014;**11**(10):e1001751.

18. Ye Z, Sharp SJ, Burgess S, et al. Association between circulating 25-hydroxyvitamin D and incident type 2 diabetes: a mendelian randomisation study. *Lancet Diabetes Endocrinol* 2015;**3**(1):35-42.

19. Brondum-Jacobsen P, Benn M, Afzal S, Nordestgaard BG. No evidence that genetically reduced 25-hydroxyvitamin D is associated with increased risk of ischaemic heart disease or myocardial infarction: a Mendelian randomization study. *Int J Epidemiol* 2015;**44**(2):651-61.

20. Kunutsor SK, Burgess S, Munroe PB, Khan H. Vitamin D and high blood pressure: causal association or epiphenomenon? *Eur J Epidemiol* 2014;**29**(1):1-14.

21. Manousaki D, Mokry LE, Ross S, Goltzman D, Richards JB. Mendelian Randomization Studies Do Not Support a Role for Vitamin D in Coronary Artery Disease. *Circ Cardiovasc Genet* 2016;**9**(4):349-56.

22. Skaaby T, Husemoen LL, Martinussen T, et al. Vitamin D status, filaggrin genotype, and cardiovascular risk factors: a Mendelian randomization approach. *PLoS One* 2013;**8**(2):e57647.

23. Vimaleswaran KS, Cavadino A, Berry DJ, et al. Association of vitamin D status with arterial blood pressure and hypertension risk: a mendelian randomisation study. *Lancet Diabetes Endocrinol* 2014;**2**(9):719-29.

24. Mokry LE, Ross S, Ahmad OS, et al. Vitamin D and Risk of Multiple Sclerosis: A Mendelian Randomization Study. *PLoS Med* 2015;**12**(8):e1001866.

25. Rhead B, Baarnhielm M, Gianfrancesco M, et al. Mendelian randomization shows a causal effect of low vitamin D on multiple sclerosis risk. *Neurol Genet* 2016;**2**(5):e97.

26. Viatte S, Yarwood A, McAllister K, et al. The role of genetic polymorphisms regulating vitamin D levels in rheumatoid arthritis outcome: a Mendelian randomisation approach. *Ann Rheum Dis* 2014;**73**(7):1430-3.

27. Yarwood A, Viatte S, Plant D, et al. Testing the role of vitamin D in response to antitumour necrosis factor alpha therapy in a UK cohort: a Mendelian randomisation approach. *Ann Rheum Dis* 2014;**73**(5):938-40.

28. Jorde R, Mathiesen EB, Rogne S, et al. Vitamin D and cognitive function: The Tromso Study. *J Neurol Sci* 2015;**355**(1-2):155-61.

29. Kueider AM, Tanaka T, An Y, et al. State- and trait-dependent associations of vitamin-D with brain function during aging. *Neurobiol Aging* 2016;**39**:38-45.

30. Mokry LE, Ross S, Morris JA, Manousaki D, Forgetta V, Richards JB. Genetically decreased vitamin D and risk of Alzheimer disease. *Neurology* 2016;**87**(24):2567-74.

31. Afzal S, Brondum-Jacobsen P, Bojesen SE, Nordestgaard BG. Genetically low vitamin D concentrations and increased mortality: Mendelian randomisation analysis in three large cohorts. *BMJ* 2014;**349**:g6330.

32. Davies JR, Field S, Randerson-Moor J, et al. An inherited variant in the gene coding for vitamin D-binding protein and survival from cutaneous melanoma: a BioGenoMEL study. *Pigment Cell Melanoma Res* 2014;**27**(2):234-43.

33. Trummer O, Pilz S, Hoffmann MM, et al. Vitamin D and mortality: a Mendelian randomization study. *Clin Chem* 2013;**59**(5):793-7.

34. Ong JS, Cuellar-Partida G, Lu Y, et al. Association of vitamin D levels and risk of ovarian cancer: a Mendelian randomization study. *Int J Epidemiol* 2016;**45**(5):1619-30.

35. Theodoratou E, Palmer T, Zgaga L, et al. Instrumental variable estimation of the causal effect of plasma 25-hydroxy-vitamin D on colorectal cancer risk: a mendelian randomization analysis. *PLoS One* 2012;**7**(6):e37662.

36. Dudding T, Thomas SJ, Duncan K, Lawlor DA, Timpson NJ. Re-Examining the Association between Vitamin D and Childhood Caries. *PLoS One* 2015;**10**(12):e0143769.

37. Li SS, Gao LH, Zhang XY, et al. Genetically Low Vitamin D Levels, Bone Mineral Density, and Bone Metabolism Markers: a Mendelian Randomisation Study. *Sci Rep* 2016;**6**:33202.

38. Tyrrell J, Richmond RC, Palmer TM, et al. Genetic Evidence for Causal Relationships Between Maternal Obesity-Related Traits and Birth Weight. JAMA 2016;**315**(11):1129-40.

39. Vimaleswaran KS, Berry DJ, Lu C, et al. Causal relationship between obesity and vitamin D status: bi-directional Mendelian randomization analysis of multiple cohorts. *PLoS Med* 2013;**10**(2):e1001383.

40. Hysinger EB, Roizen JD, Mentch FD, et al. Mendelian randomization analysis demonstrates that low vitamin D is unlikely causative for pediatric asthma. *J Allergy Clin Immunol* 2016;**138**(6):1747-49.e4.

41. Liefaard MC, Ligthart S, Vitezova A, et al. Vitamin D and C-Reactive Protein: A Mendelian Randomization Study. *PLoS One* 2015;**10**(7):e0131740.

42. Ooi EM, Afzal S, Nordestgaard BG. Elevated remnant cholesterol in 25-hydroxyvitamin D deficiency in the general population: Mendelian randomization study. *Circ Cardiovasc Genet* 2014;**7**(5):650-8.

43. Dudding T, Johansson M, Thomas SJ, Brennan P, Martin RM, Timpson NJ. Assessing the causal association between 25-hydroxyvitamin D and the risk of oral and oropharyngeal cancer using Mendelian randomization. *Int J Cancer* 2018;**143**(5):1029-36.

44. Chandler PD, Tobias DK, Wang L, et al. Association between vitamin D genetic risk score and cancer risk in a large cohort of U.S. women. *Nutrients* 2018;**10 (1)**.

45. Chen C, Zhai H, Cheng J, et al. Causal link between vitamin D and total testosterone in men: A mendelian randomization analysis. *J Clin Endocrinol Metab* 2019;**21**.

46. Larsson SC, Melhus H, Michaelsson K. Circulating Serum 25-Hydroxyvitamin D Levels and Bone Mineral Density: Mendelian Randomization Study. *J Bone Miner Res* 2018;**33**(5):840-44.

47. Sun JY, Zhao M, Hou Y, et al. Circulating serum vitamin D levels and total body bone mineral density: A Mendelian randomization study. *J Cell Mol Med* 2019;**23**(3):2268-71.

48. Dimitrakopoulou VI, Tsilidis KK, Haycock PC, et al. Circulating vitamin D concentration and risk of seven cancers: Mendelian randomisation study. *BMJ (Clinical research ed)* 2017;**359**:j4761.

49. Sun YQ, Brumpton BM, Bonilla C, et al. Serum 25-hydroxyvitamin D levels and risk of lung cancer and histologic types: A Mendelian randomisation analysis of the HUNT study. *Eur Respir J* 2018;**51**(6).

50. Ong JS, Gharahkhani P, An J, et al. Vitamin D and overall cancer risk and cancer mortality: a Mendelian randomization study. *Hum Mol Genet* 2018;**27**(24):4315-22.

51. Aspelund T, Grubler MR, Smith AV, et al. Effect of genetically low 25-hydroxyvitamin D on mortality risk: Mendelian randomization analysis in 3 large european cohorts. *Nutrients* 2019;**11 (1)**.

52. Gianfrancesco MA, Stridh P, Rhead B, et al. Evidence for a causal relationship between low Vitamin D, high BMI, and pediatric-onset MS. *Neurology* 2017;**88**(17):1623-29.

53. He Y, Timofeeva M, Farrington SM, et al. Exploring causality in the association between circulating 25-hydroxyvitamin D and colorectal cancer risk: A large Mendelian randomisation study. *BMC Med* 2018;**16 (1)**.

54. Cuellar-Partida G, Williams KM, Yazar S, et al. Genetically low vitamin D concentrations and myopic refractive error: A Mendelian randomization study. *Int J Epidemiol* 2017;**46**(6):1882-90.

55. Winslow UC, Nordestgaard BG, Afzal S. High plasma 25-hydroxyvitamin D and high risk of nonmelanoma skin cancer: a Mendelian randomization study of 97 849 individuals. *Br J Dermatol* 2018;**178**(6):1388-95.

56. Havdahl A, Mitchell R, Paternoster L, Davey Smith G. Investigating causality in the association between vitamin D status and self-reported tiredness. *Sci Rep* 2019;**9**(1):2880.

57. Takahashi H, Cornish AJ, Sud A, et al. Mendelian randomisation study of the relationship between vitamin D and risk of glioma. *Sci Rep* 2018;**8**(1):2339.

58. Larsson SC, Traylor M, Malik R, et al. Modifiable pathways in Alzheimer's disease: Mendelian randomisation analysis. *BMJ* 2017;**359**:j5375.

59. Teumer A, Gambaro G, Corre T, et al. Negative effect of Vitamin D on kidney function: A Mendelian randomization study. *Nephrol Dial Transplant* 2018;**33**(12):2139-45.

60. Magnus MC, Miliku K, Bauer A, et al. Vitamin D and risk of pregnancy related hypertensive disorders: Mendelian randomisation study. *BMJ (Online)* 2018;**361** (k2167).

61. Bowman K, Jones L, Pilling LC, et al. Vitamin D levels and risk of delirium: A mendelian randomization study in the UK Biobank. *Neurology* 2019;**92**(12):e1387-e94.

62. Bae SC, Lee YH. Vitamin D level and risk of systemic lupus erythematosus and rheumatoid arthritis: a Mendelian randomization. *Clin Rheumatol* 2018;**37**(9):2415-21.

63. Magnus MC, Miliku K, Bauer A, et al. Vitamin D and Risk of Pregnancy-Related Hypertensive Disorders: Mendelian Randomization Study. *BMJ* 2018;**361**:k2167.

64. Wang N, Chen C, Zhao L, et al. Vitamin D and Nonalcoholic Fatty Liver Disease: Bi-directional Mendelian Randomization Analysis. *EBioMedicine* 2018;**28**:187-93.

65. Lund-Nielsen J, Vedel-Krogh S, Kobylecki CJ, Brynskov J, Afzal S, Nordestgaard BG. Vitamin D and Inflammatory Bowel Disease: Mendelian Randomization Analyses in the Copenhagen Studies and UK Biobank. *J Clin Endocrinol Metab* 2018;**103**(9):3267-77.

66. Larsson SC, Traylor M, Markus HS, Michaelsson K. Serum parathyroid hormone, 25-hydroxyvitamin D, and risk of Alzheimer's disease: A mendelian randomization study. *Nutrients* 2018;**10 (9)** (1243).

67. Michaelsson K, Melhus H, Larsson SC. Serum 25-hydroxyvitamin D concentrations and major depression: A mendelian randomization study. *Nutrients* 2018;**10 (12)**.

68. Larsson SC, Traylor M, Mishra A, Howson JMM, Michaelsson K, Markus HS. Serum 25-hydroxyvitamin D concentrations and ischemic stroke and its subtypes a Mendelian randomization study. *Stroke* 2018;**49**(10):2508-11.

69. Tan VY, Biernacka KM, Dudding T, et al. Reassessing the association between circulating Vitamin D and IGFBP-3: Observational and Mendelian randomization estimates from independent sources. *Cancer Epidemiol Biomarkers Prev* 2018;**27**(12):1462-71.

70. Mai XM, Videm V, Sheehan NA, Chen Y, Langhammer A, Sun YQ. Potential causal associations of serum 25-hydroxyvitamin D with lipids: a Mendelian randomization approach of the HUNT study. *Eur J Epidemiol* 2019;**34**(1):57-66.

71. Larsson SC, Singleton AB, Nalls MA, Richards JB. No clear support for a role for vitamin D in Parkinson's disease: A Mendelian randomization study. *Mov Disord* 2017;**32**(8):1249-52.

72. Noordam R, Hamer MA, Pardo LM, et al. No Causal Association between 25-Hydroxyvitamin D and Features of Skin Aging: Evidence from a Bidirectional Mendelian Randomization Study. *J Invest Dermatol* 2017;**137**(11):2291-97.

73. Dong J, Gharahkhani P, Chow WH, et al. No Association Between Vitamin D Status and Risk of Barrett's Esophagus or Esophageal Adenocarcinoma-a Mendelian Randomization Study. *Clin Gastroenterol Hepatol* 2019;**01**.

74. Lu L, Bennett DA, Millwood IY, et al. Association of vitamin D with risk of type 2 diabetes: A Mendelian randomisation study in European and Chinese adults. *PLoS Med* 2018;**15 (5)** (e1002566).
